# Supplementary material for: HLA alleles, disease severity, and age associate with T-cell responses following infection with SARS-CoV-2
Source: Commun Biol. 2022 Sep 6;5:914. doi: 10.1038/s42003-022-03893-w (PMC9446630; doi:10.1038/s42003-022-03893-w)
Supplement: Supplementary file 2 — Supplementary Information [file 42003_2022_3893_MOESM2_ESM.pdf]

Supplementary figures and tables:

HLA alleles, disease severity and age associate with T-cell responses following infection with SARS-CoV-2

Thorunn A. Olafsdottir[1,9*], Kristbjorg Bjarnadottir[1,9], Gudmundur L. Norddahl[1], Gisli H. Halldorsson[1], Pall Melsted[1,2], Kristbjorg Gunnarsdottir[1], Erna Ivarsdottir[1], Thorhildur Olafsdottir[1], Asgeir O. Arnthorsson[1], Fannar Theodors[1], Elias Eythorsson[3], Dadi Helgason[3], Hannes P. Eggertsson[1], Gisli Masson[1], Sólveig Bjarnadottir[3,4], Saedis Saevarsdottir[1,4,5], Hrafnhildur L. Runolfsdottir[3], Isleifur Olafsson[6], Jona Saemundsdottir[1], Martin I. Sigurdsson[4,7], Ragnar F. Ingvarsson[3], Runolfur Palsson[3,4], Gudmundur Thorgeirsson[1,4], Bjarni V. Halldorsson[1,8], Hilma Holm[1], Mar Kristjansson[3], Patrick Sulem[1], Unnur Thorsteinsdottir[1,4], Ingileif Jonsdottir[1,4], Daniel F. Gudbjartsson[1,2], Kari Stefansson[1,4,*]

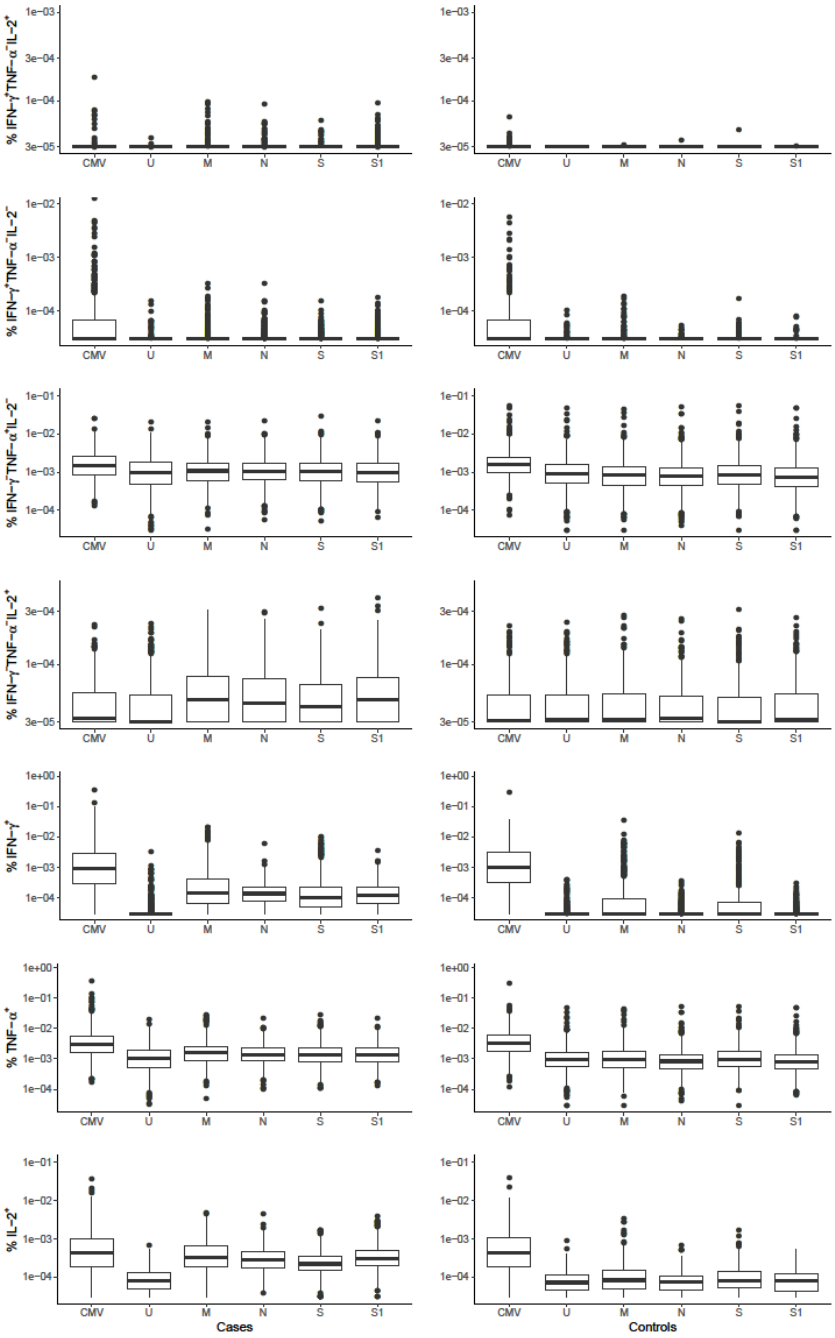

Supplementary Figure 1. Box plots summarizing the frequencies of CD4$^+$ T-cell functional phenotype CD4$^+$ T-cell responses induced by the positive control (CMV), unstimulated control (U), and the four different SARS-CoV-2 peptide pools (M, N, S and S1) in SARS-CoV-2 cases (n=767) and non-infected controls (n=392). All IFN-γ^+, all IL-2$^+$, all TNF-α^+ as well as single positive (IFN-γ^+, IL-2$^+$ or TNF-α^+) and double positive CD4$^+$ T-cells are shown. The bottom and top of the boxes correspond to the 25th (Q1) and 75th (Q3) percentiles, the line inside the box corresponds to the median, and the whiskers are located at max(min(Expression), Q1 – 1.5 IQR) and min(max(Expression), Q3 + 1.5 IQR[where IQR is

the interquartile range = Q3 – Q1]), respectively. The error bars indicate 95% confidence intervals (CI).

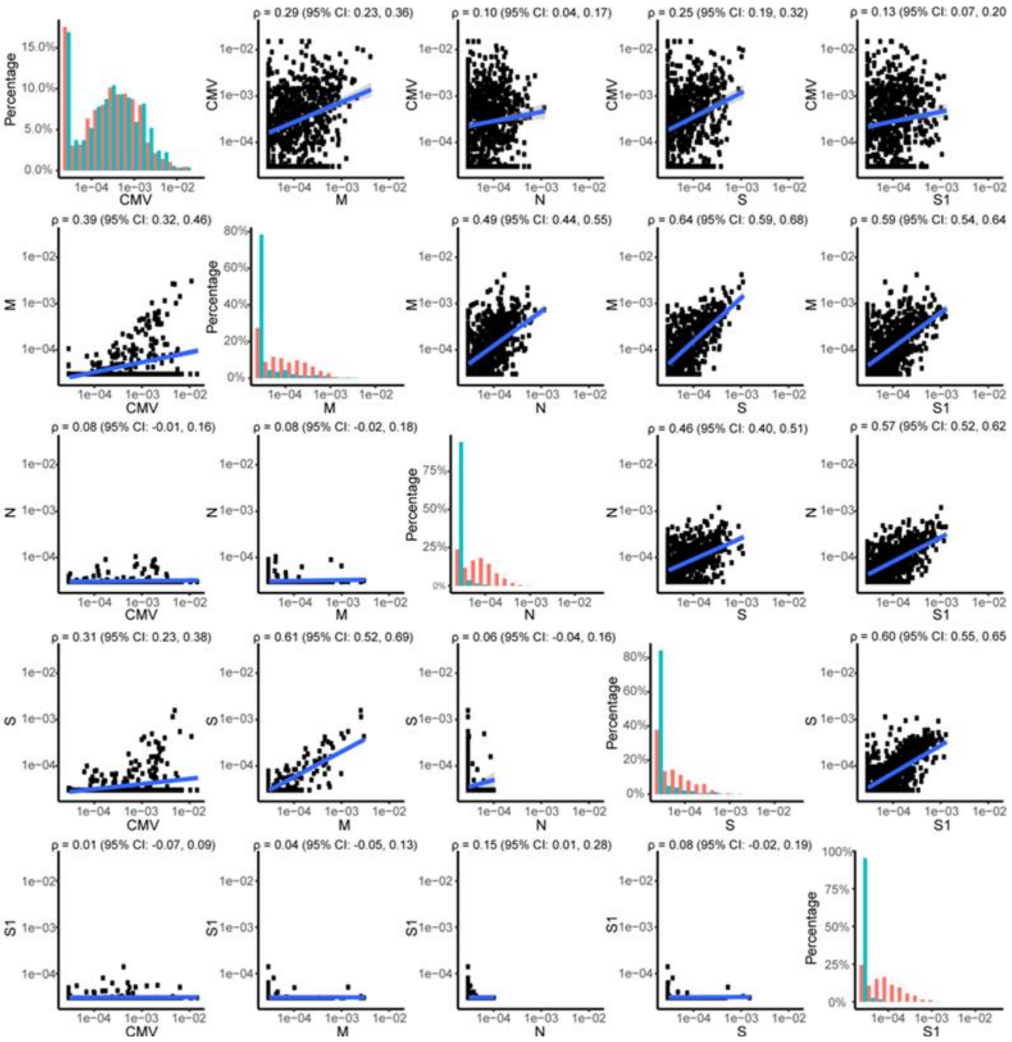

Supplementary Figure 2. Polyfunctional SARS-CoV-2 reactive CD4$^+$ T-cell responses discriminate cases from controls. Column plots show cases in red (n=767) and controls (n=392) in blue where the Y-axes show % of individuals in each dataset and the X- axes show frequency of polyfunctional (IFN-γ^+TNF-α^+IL-2$^+$) CD4$^+$ T-cells reacting to CMV, M, N, S or S1 stimulations. The scatter plots on the right of the diagonal show correlation of frequencies of polyfunctional CD4$^+$ T-cells within the total CD4$^+$ T-cell population for the different stimulations (as indicated on each plot) in cases and the scatter plots on the left show the same for uninfected controls. The solid blue line indicates the logistic-regression line and the grey area around the blue line indicates the 95% confidence intervals (CI).The different stimulation conditions are: the positive control (CMV) and the four different SARS-CoV-2 proteins (M, N, S and S1). Correlation between T-cell responses was estimated using Spearman's

correlation coefficient and a Jackknife method was used to calculate confidence intervals. Spearman correlation (ρ) with 95% CI is given for each scatter plot.

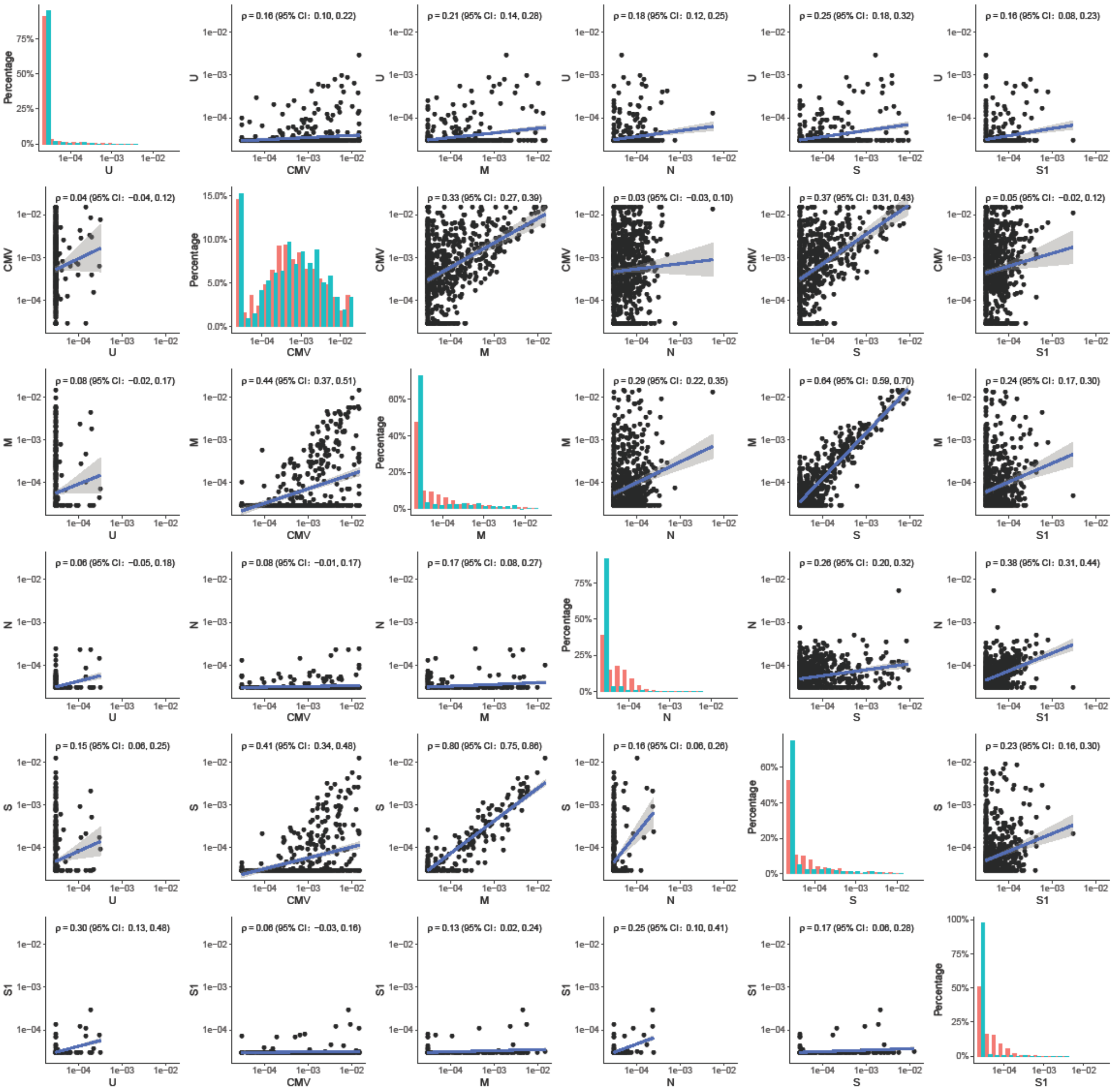

Supplementary Figure 3. IFN-γ$^+$TNF-α$^+$ double positive CD4$^+$ T-cells in SARS-CoV-2 cases and uninfected controls. Column plots show cases in red (n=767) and controls in blue (n=392) where the Y-axes shows % of individuals in each dataset and the X-axes shows frequency of double cytokine producing (IFN-γ$^+$TNF-α$^+$) CD4$^+$ T-cells reacting to no stimulation (U), CMV, M, N, S or S1 stimulation. The scatter plots on the right show correlation of the different stimulations (as indicated on each plot) in cases and the scatter plots on the left show the same for non-infected controls. The solid blue line indicates the logistic-regression line and the grey area around the blue line indicates the 95% confidence intervals (CI). Spearman correlation (ρ) with 95% CI is given for each scatter plot.

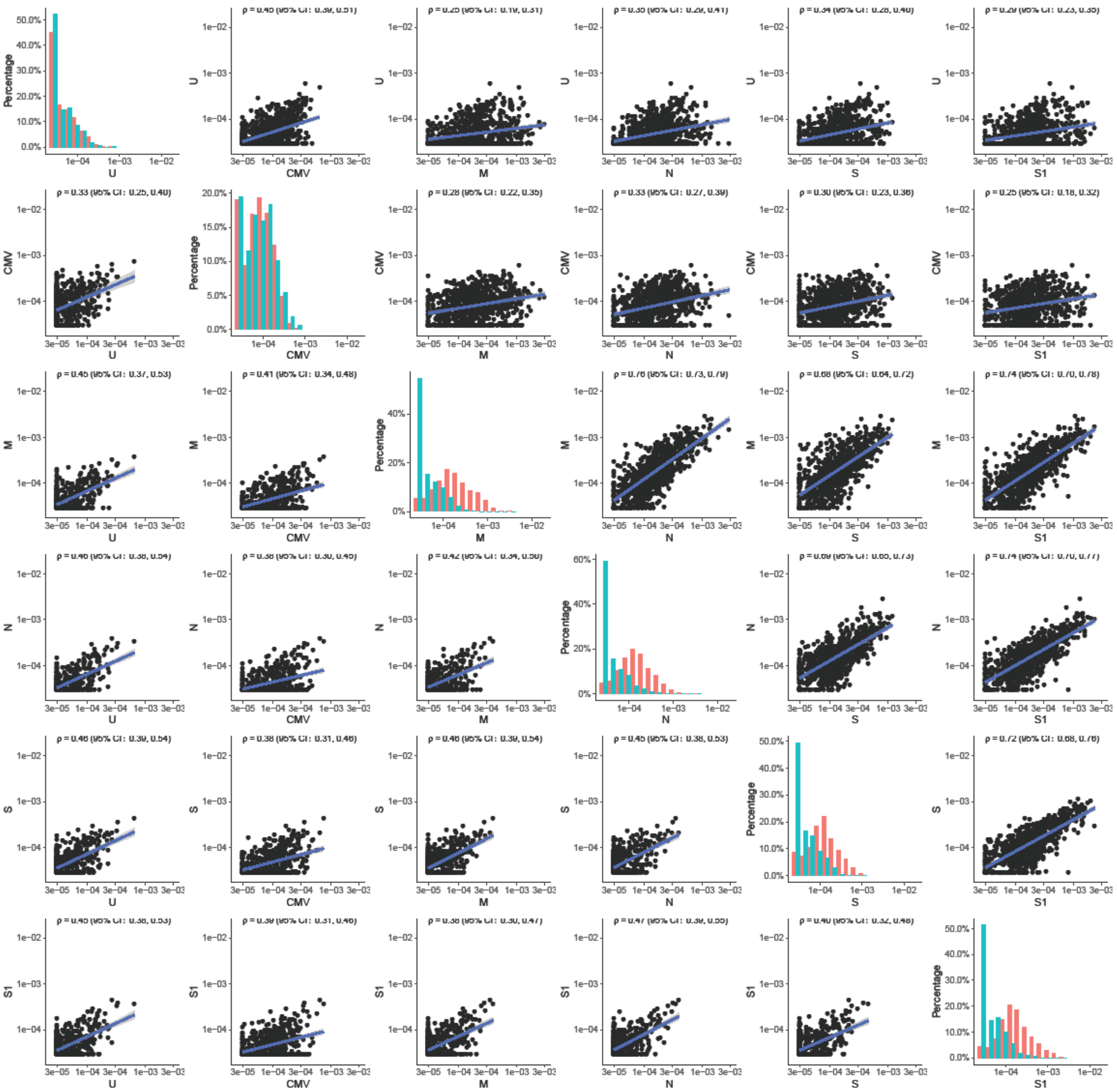

Supplementary Figure 4. IL-2^{+}TNF-α^{+} double positive CD4^{+} T-cells in SARS-CoV-2 cases and uninfected controls. Column plots show cases in red (n=767) and controls in blue (n=392) where the Y-axes shows % of individuals in each dataset and the X-axes shows frequency of double cytokine producing (IL-2^{+}TNF-α^{+}) CD4^{+} T-cells reacting to no stimulation (U), CMV, M, N, S or S1 stimulation. The scatter plots on the right show correlation of the different stimulations (as indicated on each plot) in cases and the scatter plots on the left show the same for non-infected controls. The solid blue line indicates the logistic-regression line and the grey area around the blue line indicates the 95% confidence intervals (CI). Spearman correlation (ρ) with 95% CI is given for each scatter plot.

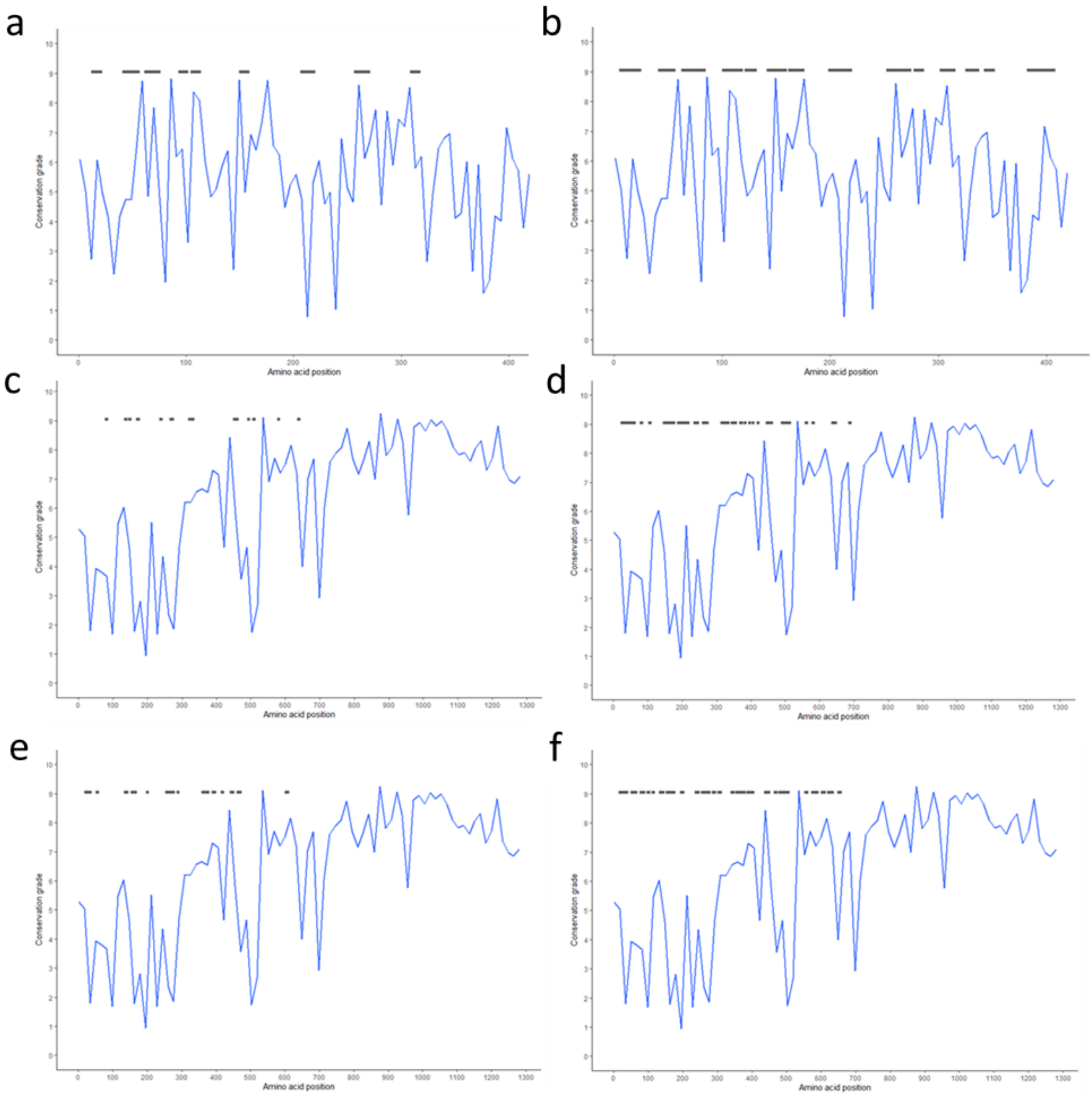

Supplementary Figure 5 Conservation of SARS-CoV-2 S and N protein derived peptides predicted to bind HLA alleles associating with CD8$^+$ T-cell responses. Multiple sequence alignment was generated by the ConSurf algorithm (https://consurf.tau.ac.il) using the structure of chain A of the SARS-CoV-2 spike protein in the closed state (PDB ID 6VXX) as a reference, whereas the amino acid sequence was used for the N protein as a reference in absence of a structure of the whole protein. The figures show conservation of the N (a and b) and S (c-f) protein sequence on the y-axes and amino acid position on the x-axes. Amino acid positions of the peptides predicted to bind to each HLA allele are shown as grey lines above each plot. (a) Peptides from the N protein predicted to bind strongly to HLA-B*07:02. (b) Peptides from the N protein predicted to bind weakly to HLA-B*07:02. (c) Peptides from the

S1 subunit (predicted to bind strongly to HLA-C*07:02. (d) Peptides from the S1 subunit predicted to bind weakly to HLA-C*07:02. (e) Peptides from the S1 subunit predicted to bind strongly to HLA-A*01:01. (f) Peptides from the S1 subunit predicted to bind weakly to HLA-A*01:01. Further information on the peptides can be found in table S5 .

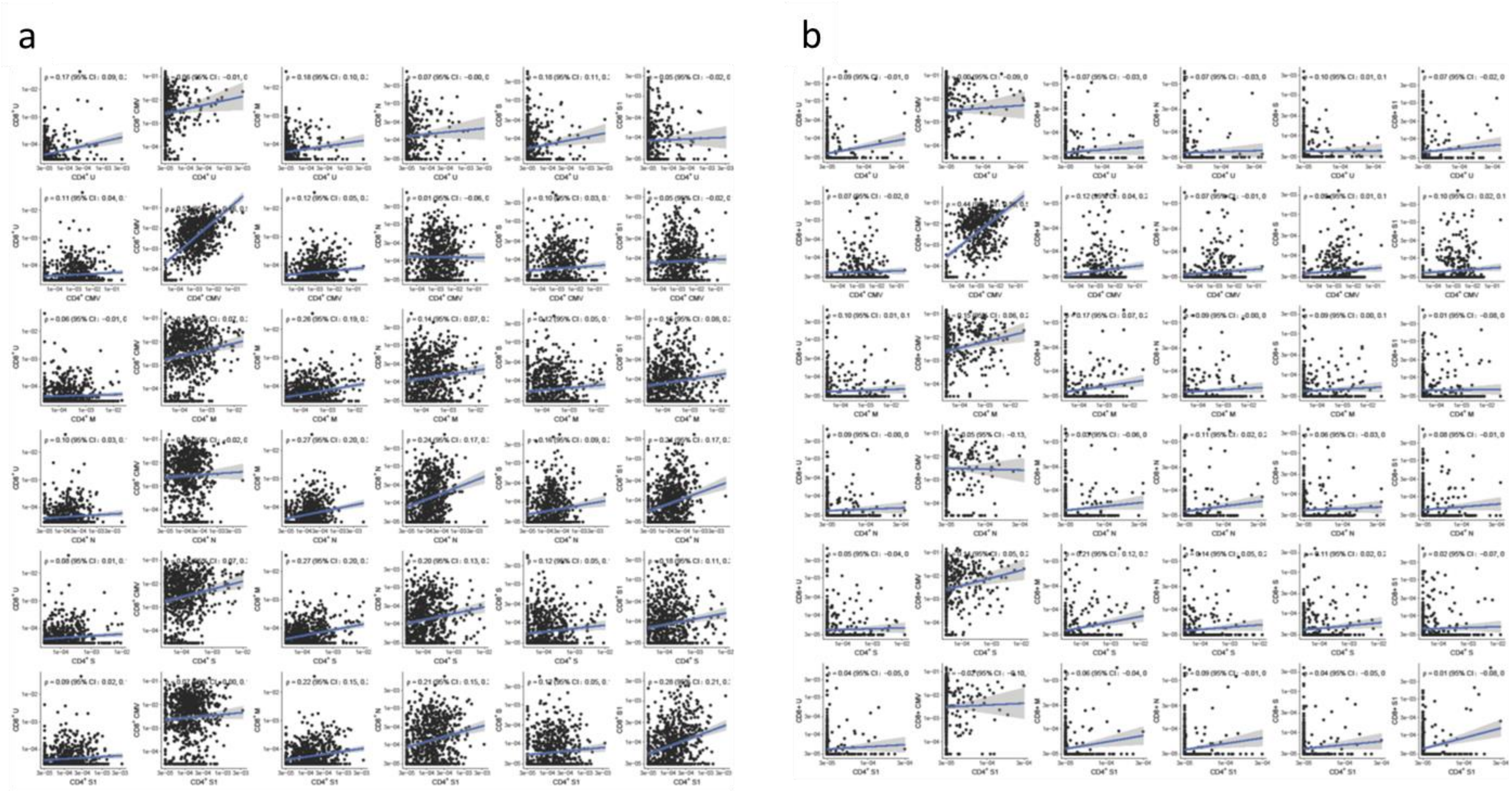

Supplementary Figure 6. Correlation between SARS-CoV-2 reactive CD4$^+$ and CD8$^+$ T-cell responses. The scatter plots show correlation between polyfunctional CD4$^+$ and all IFN-γ^+ CD8$^+$ T-cell responses of the different stimulations (as indicated on each plot) in (a) cases (CD4$^+$ n=767 and CD8$^+$ n=764) and (b) non-infected controls (CD4$^+$ n=392 and CD8$^+$ n=387). Spearman correlation (ρ) with 95% confidence intervals

(CI) is given for each scatter plot. The solid blue line indicates the logistic-regression line and the grey area around the blue line indicates the 95% CI.

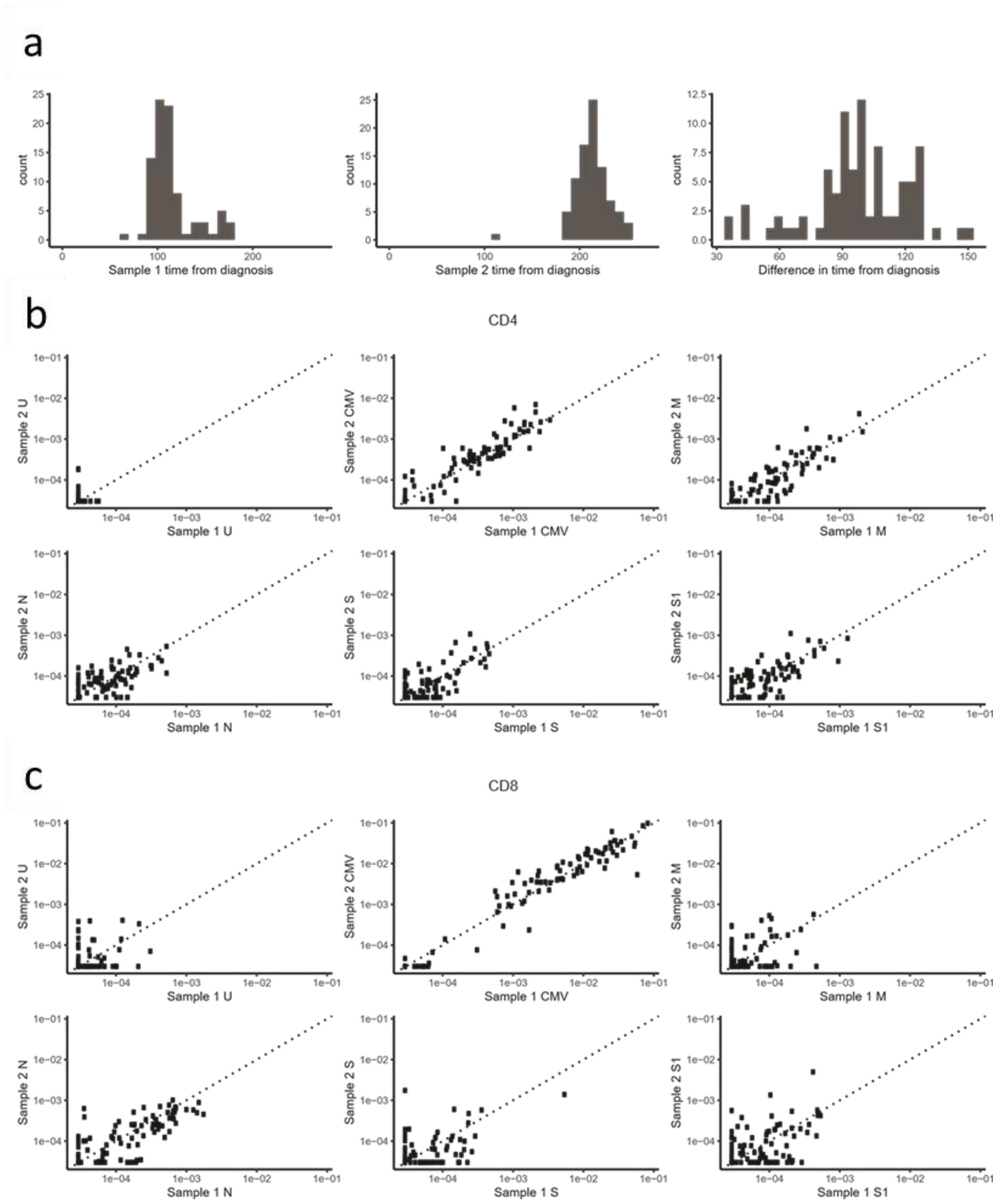

Supplementary Figure 7. Kinetics of the SARS-CoV-2 induced T-cell responses in the first eight months after diagnosis. (A) Overview of 90 paired samples from the same individuals collected 3-6 months from SARS-CoV-2 diagnosis (sample 1) and again 3-5 months later (sample 2). Correlation between sample 1 and sample 2 for frequency of (B) polyfunctional (IFN-γ^+TNF-α^+IL-2^+) SARS-CoV-2 reactive $CD4^+$ T-cells and (C) all IFN-γ^+ $CD8^+$ T-cells.

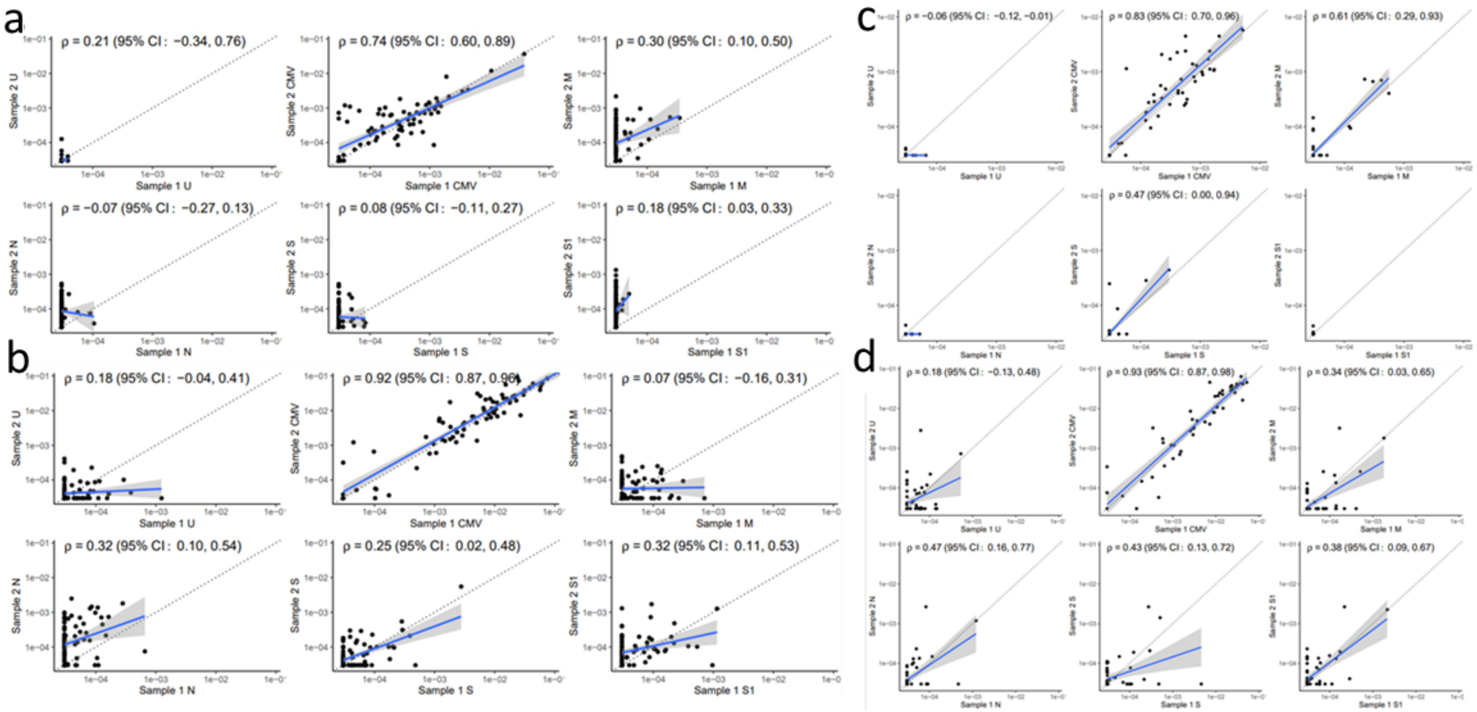

Supplementary Figure 8. Kinetics of the T-cell responses in paired samples. Overview of paired samples from the same individuals collected before March 2020 (sample 1) and during the pandemic (sample 2) in Iceland in (a-b) 80 cases and (c-d) 44 controls. Correlation of (a) polyfunctional $CD4^+$ and (b) all IFN-γ^+ $CD8^+$ T-cell responses in paired samples from SARS-CoV-2 cases. Correlation of (c) polyfunctional $CD4^+$ and (d) all IFN-γ^+ $CD8^+$ T-cell responses in paired samples from uninfected controls. The solid blue line indicates the logistic-regression line and the grey area around the blue line indicates the 95% confidence intervals (CI).

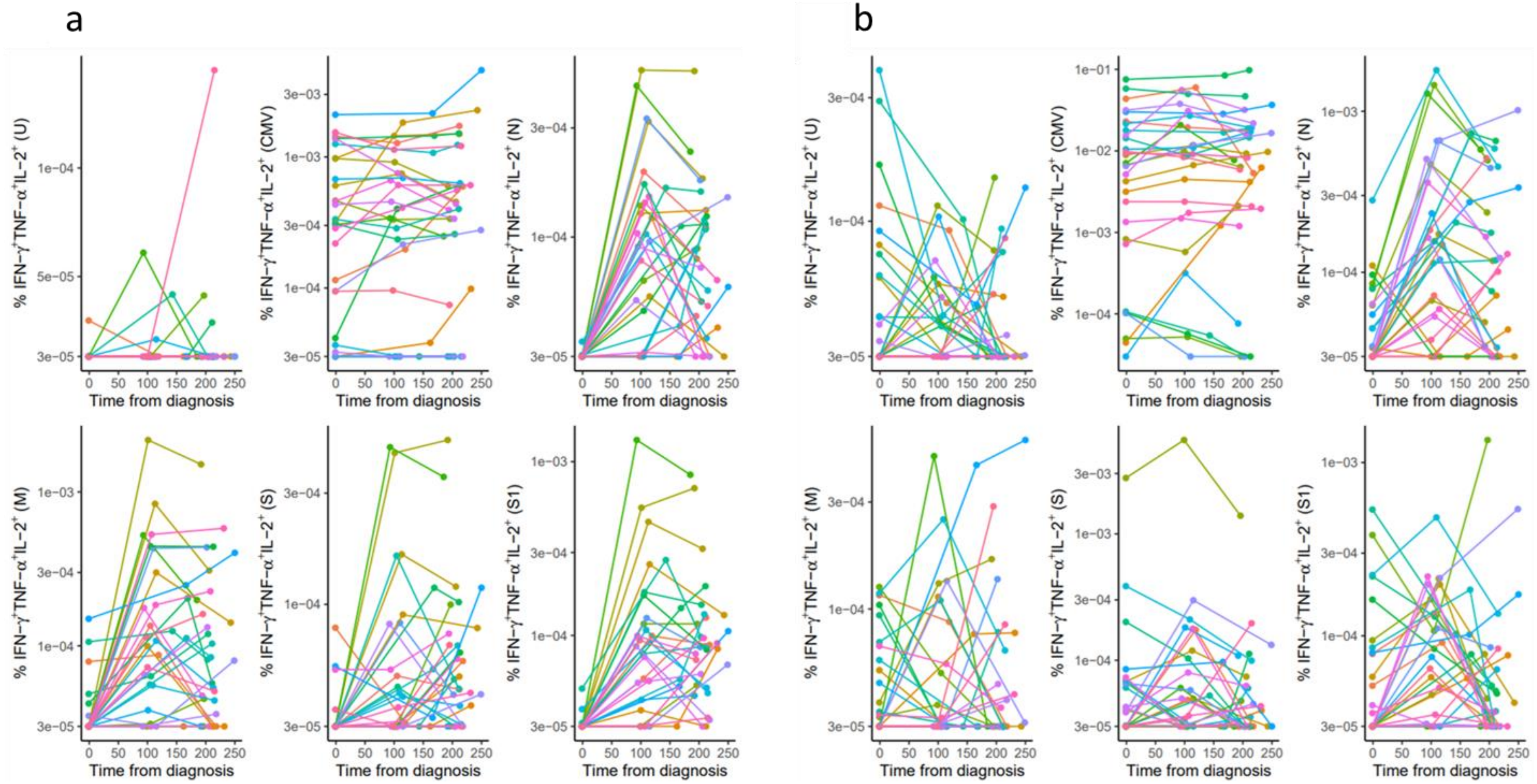

Supplementary Figure 9. Kinetics of SARS-CoV-2 reactive polyfunctional CD4$^+$ and CD8$^+$ T-cell responses Samples from 30 SARS-CoV-2 cases where the first sample was collected pre-pandemic (labelled as 0) and then two samples were obtained after diagnosis of the infection. Time from diagnosis is given in days on the X-axis, Y-axes shows percent of (a) polyfunctinal CD4$^+$ and (b) CD8$^+$ T-cells within the CD4$^+$ and CD8+ T-cell population, respectively.

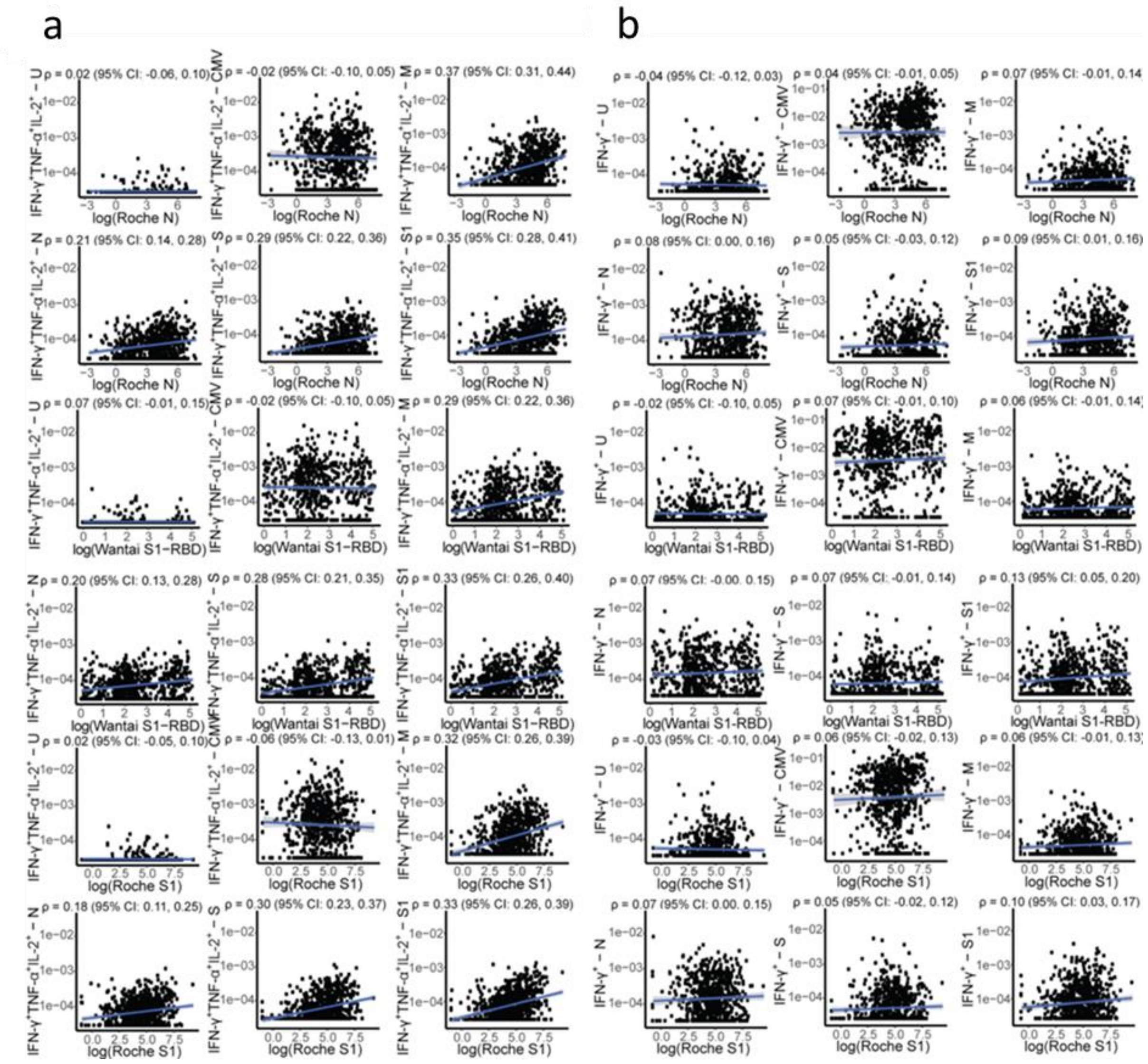

Supplementary Figure 10 SARS-CoV-2 reactive CD4^{+} but not CD8^{+} T-cell responses correlate with total antibody levels directed against S1-RBD and N proteins. Scatter plot show correlation between (a) polyfunctional CD4^{+} as well as (b) all IFN-γ^{+} CD8^{+} T-cells and humoral responses. Antibody responses were measured using pan-Ig assays detecting IgM, IgG and IgA against the N protein (Roche) and the RBD of the S1 subunit (Wantai and Roche) as indicated on the x-axes. Antibody titers are given on log scale on the X-axes and percentage (a) polyfunctional CD4^{+} T-cells out of all CD4^{+} T-cells and (b) all IFN-γ^{+} CD8^{+} T-cells out of all CD8^{+} T-cells are given on the Y-axes (n=459 convalescent SARS-CoV-2 infected individuals). The solid blue line indicates the logistic-regression line and the grey area around the blue line indicates the 95% confidence intervals (CI).

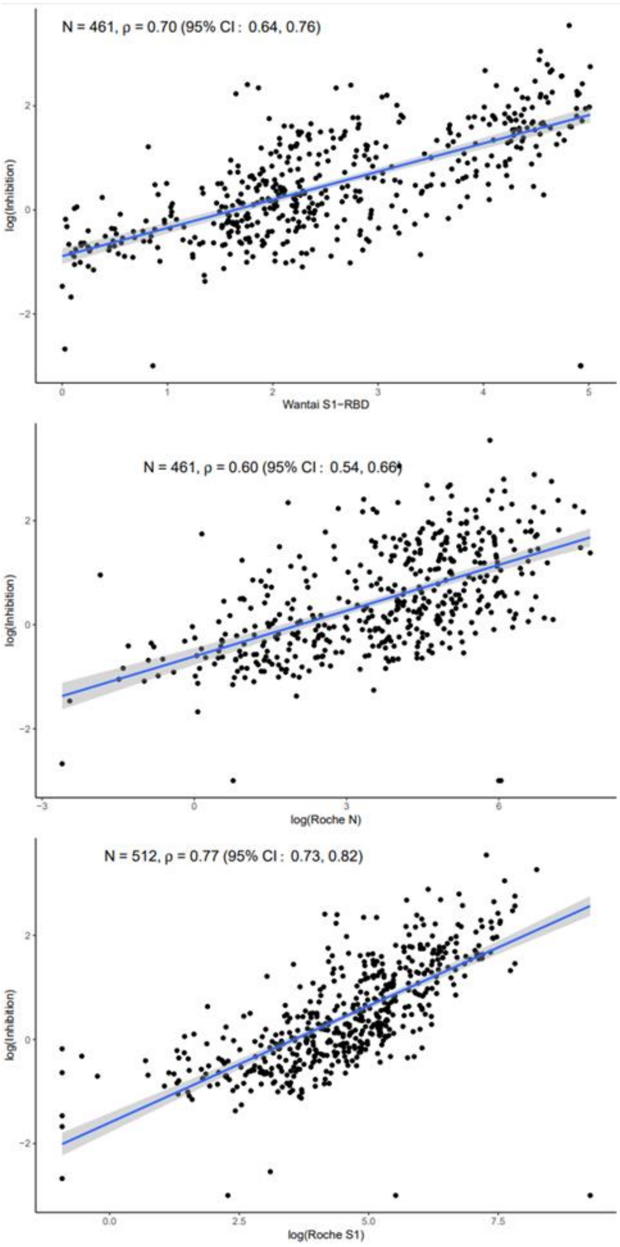

Supplementary Figure 11 Correlation between pan-Ig and ACE2 blocking assays Scatter plot show correlation between inhibition capacity of the antibodies measured using an MSD-ECLIA based ACE2 blocking assay and pan-Ig directed against S1-RBD measured with Wantai (top figure) or Roche (bottom figure) or against N measured with Roche (middle figure). Inhibition efficacy of the antibodies is given on log scale on the Y-axes and pan-Ig levels are given on the Y-axes as indicated. The solid blue line indicates the logistic-regression line and the grey area around the blue line indicates the 95% confidence intervals (CI). Measurements were performed on subset of SARS-CoV-2 convalescent cases as indicated on the figure.

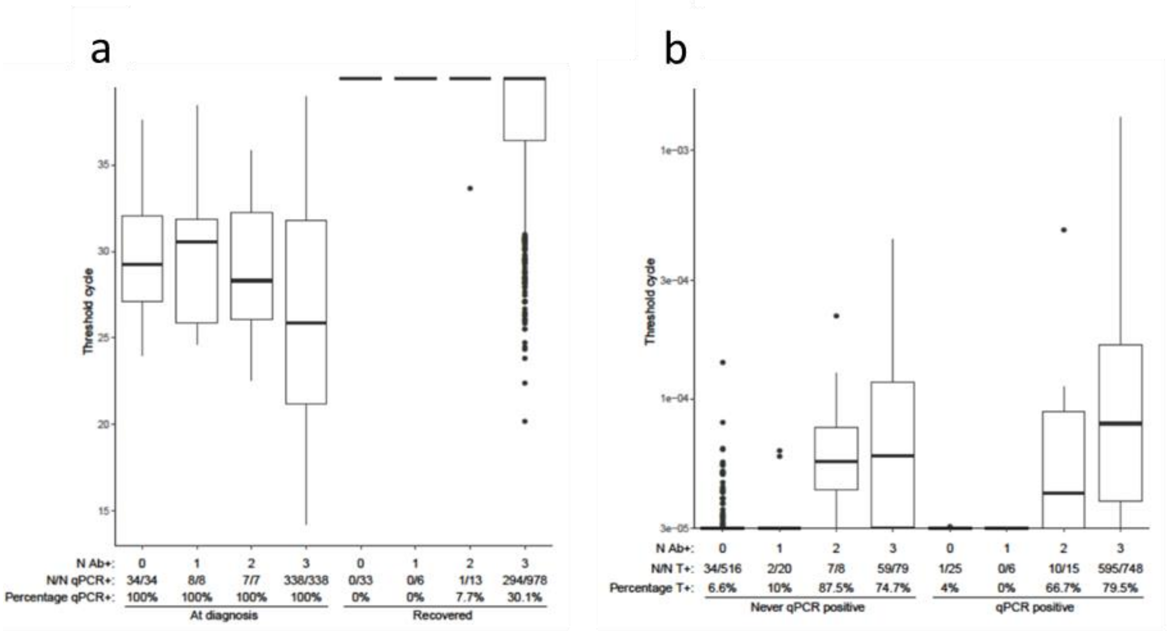

Supplementary Figure 12. qPCR threshold cycles in cases at diagnosis and following recovery as well as those that were qPCR negative compared with those that tested qPCR positive, shown in relation to seropositivity and T cell responses. Recovered samples were measured 2-6 weeks after qPCR-confirmed SARS-CoV-2 diagnosis. N Ab+ refers to the number of samples that were seropositive when measured by three different pan-Ig (IgM, IgG and IgA) against the N protein (Roche ECLIA) and RBD of the S1 subunit (Wantai ELISA and Roche ECLIA). (a) Number and percentage of qPCR-positive individuals. (b) Number and percentage of individuals with detectable (above 0.001%) $CD4^+$ T-cell responses against the N, M, S or S1 proteins. The bottom and top of the boxes correspond to the 25th (Q1) and 75th (Q3) percentiles, the line inside the box corresponds to the median, and the whiskers are located at max(min(Expression), Q1 – 1.5 IQR) and min(max(Expression), Q3 + 1.5 IQR [where IQR is the interquartile range = Q3 – Q1]), respectively.

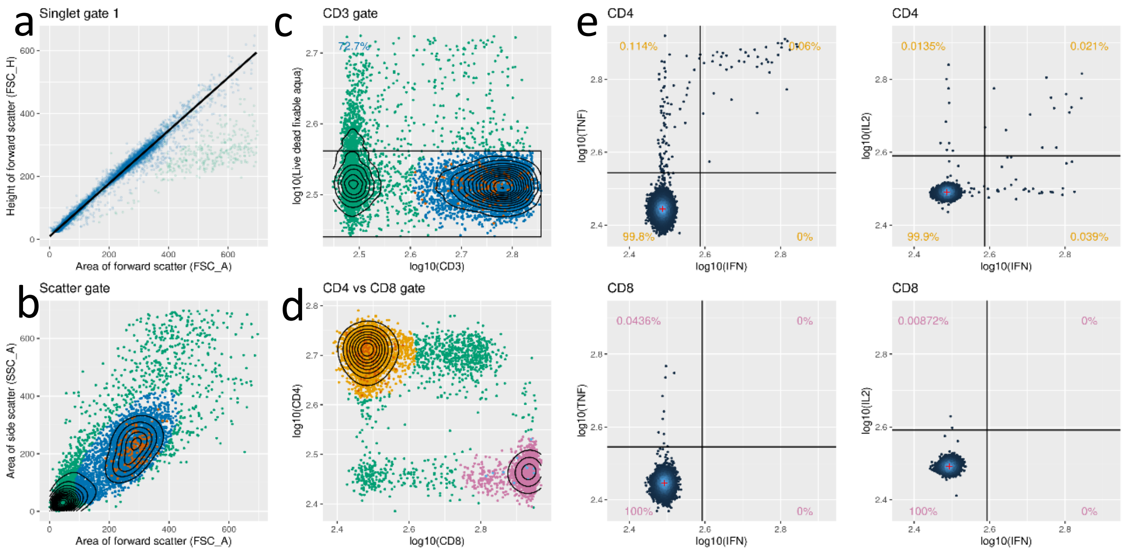

Supplementary Figure 13: Flow cytometry gating in a sample stimulated with S protein taken from a SARS-CoV-2 positive subject. Gating was processed using custom algorithm shown in panels (a) to (d). (a) *Singlet gate*; Cells classified as singlets in dark-blue and doublets cells in green were filtered out. The black line corresponds to the last line fit in the singlet gate. (b) *Scatter gate*; Lymphocytes labeled in dark-blue or if the CD4^{+} lymphocytes cells were positive for any of the three tested cytokines then they were labeled as orange color, or if the CD8^{+} lymphocytes were positive for any of the three tested cytokines, they were labeled as light-blue. Green colored cells were filtered out. Black contour bands represent T-cell density (c) *CD3 gate*; Cells classified as live are shown within a black square. CD3 lymphocytes (CD3^{+}) have the same color-code as shown in scatter gate description. Green points indicate dead cells or cells other than CD3 lymphocytes and were filtered out. (d) *CD4 vs CD8 gate* CD4^{+} cells are shown in yellow and orange if they were cytokine positive. CD8^{+} cells are shown in pink and light-blue if they were cytokine positive. (e) Response of SARS-CoV-2 reactive CD4 (top) and CD8 (bottom) cells. Red cross marks the median cytokine levels. Boundary of a positive cytokine response is plotted with black lines. Percentage of single/double cytokine positive or negative cells is marked in each quadrant corner.

Supplementary Table 1. Effect of age and sex on $CD4^+$ and $CD8^+$ T cell counts. SARS-CoV-2 cases (n=759), females (n=405) and males n=354). Uninfected controls (n=491), females (n=290) and males (n=201). Spearman's correlation coefficient was used to estimate correlation between counts of T-cell subsets and age/sex and a Jackknife method was used to calculate confidence intervals. Significance was assessed with the standard linear regression t-test with $\boldsymbol{n}-\boldsymbol{p}$ degrees of freedom, where $\boldsymbol{n}$ is the number of available observations and $\boldsymbol{p}$ is the number of parameters (2 or 3).

	**Model**	**Age**		**Female sex**		**Difference between pre-pandemic and SARS-CoV-2 cases**		**Difference between controls and cases**	
**Cell type**		**Effect (95% CI)**	**P**	**Effect (95% CI)**	**P**	**Effect (95% CI)**	**P**	**Effect (95% CI)**	**P**
CD4	Unadjusted	-	-	-	-	-7.7% (-11.9%, -3.2%)	0.00088	-0.8% (-3.9%, 2.4%)	0.61
	Adj. for age and sex	0.1% (0%, 0.2%)	0.096	10.7% (7.6%, 13.8%)	1.5e-12	-9.0% (-13.2%, -4.5%)	0.00011	-2.3% (-5.5%, 0.9%)	0.16
CD8	Unadjusted	-	-	-	-	-10.7% (-16.3%, -4.6%)	0.00075	-5.6% (-9.7%, -1.3%)	0.011
	Adj. for age and sex	-0.6% (-0.8%, -0.5%)	3.8e-22	-4.1% (-7.7%, -0.3%)	0.033	-3.5% (-9.6%, 2.9%)	0.28	1.4% (-3.1%, 6%)	0.55

Supplementary Table 2. Effect of SARS-CoV-2 infection status and severity of COVID-19 on SARS-CoV-2 and CMV reactive CD4$^+$ T-cell responses based on GLS and GLM models. Effects represent differences in the logarithm of the fraction (GLS) and absolute count (GLM-quasi-Poisson) of stimulated cells between cases (n=767) and controls (n=392), per unit of the severity scale in cases (n=545), and between cases with the mildest disease severity (n=208) and controls (n=392). P value corrected for multiple testing P<0.05/60=8.3E-04. Significance was assessed with the standard linear regression t-test with $\boldsymbol{n-p}$ degrees of freedom, where $\boldsymbol{n}$ is the number of available observations and $\boldsymbol{p}$ is the number of parameters (2 or 3)

	Infection association (N cases=767, N controls=392)				Severity scale association (N=545)				Mild infection association (N cases=208, N controls=392)			
	GLS		GLM – quasi-Poisson		GLS		GLM – quasi-Poisson		GLS		GLM – quasi-Poisson	
Protein	Effect (95% CI)	P	Effect (95% CI)	P	Effect (95% CI)	P	Effect (95% CI)	P	Effect (95% CI)	P	Effect (95% CI)	P
**All IFN-γ producing cells**												
U	0.08 (0.02, 0.14)	0.011	0.68 (0.27, 1.09)	0.0012	-0.02 (-0.07, 0.04)	0.57	-0.18 (-0.49, 0.14)	0.27	0.1 (0.02, 0.17)	0.013	0.9 (0.42, 1.38)	0.00026
CMV	0.21 (-0.01, 0.44)	0.067	0.42 (-0.06, 0.89)	0.089	-0.05 (-0.22, 0.12)	0.56	-0.29 (-0.6, 0.01)	0.058	0.36 (0.05, 0.66)	0.021	0.78 (0.1, 1.45)	0.025
M	1.03 (0.87, 1.19)	5.7e-33	0.66 (0.27, 1.05)	0.00094	0.22 (0.09, 0.34)	0.00057	0.12 (-0.12, 0.36)	0.34	0.75 (0.53, 0.98)	1.6e-10	0.51 (-0.05, 1.06)	0.074
N	1.3 (1.21, 1.39)	9.3e-142	2.21 (1.98, 2.44)	9.3e-69	0.12 (0.05, 0.19)	0.0018	0.11 (0.03, 0.2)	0.012	1.19 (1.09, 1.28)	1.5e-99	2.09 (1.89, 2.28)	2.1e-76
S	0.75 (0.62, 0.89)	8e-27	0.72 (0.35, 1.09)	0.00015	0.15 (0.05, 0.25)	0.0051	0.11 (-0.11, 0.33)	0.33	0.55 (0.37, 0.73)	5e-9	0.53 (0.03, 1.03)	0.04
S1	1.35 (1.26, 1.44)	8.9e-151	2.69 (2.42, 2.97)	3.9e-72	0.21 (0.13, 0.28)	3.4e-7	0.32 (0.23, 0.4)	5.4e-13	1.12 (1.03, 1.2)	3.5e-102	2.26 (2.04, 2.47)	1.6e-73
**All TNF-α producing cells**												
U	0.11 (-0.01, 0.23)	0.077	-0.02 (-0.21, 0.18)	0.85	-0.12 (-0.21, -0.03)	0.013	-0.12 (-0.21, -0.02)	0.016	0.19 (0.03, 0.35)	0.018	0.1 (-0.23, 0.42)	0.56
CMV	0.12 (-0.01, 0.24)	0.07	0.24 (-0.08, 0.57)	0.14	-0.06 (-0.15, 0.04)	0.24	-0.23 (-0.46, -0.01)	0.041	0.2 (0.03, 0.37)	0.019	0.53 (0.04, 1.01)	0.034
M	0.58 (0.46, 0.69)	3.3e-22	0.36 (0.18, 0.55)	0.00016	0.08 (0, 0.16)	0.062	0.06 (-0.05, 0.16)	0.29	0.47 (0.31, 0.64)	1.6e-8	0.31 (0, 0.61)	0.049
N	0.65 (0.54, 0.75)	8.6e-33	0.32 (0.11, 0.53)	0.0028	0.03 (-0.04, 0.1)	0.47	0.01 (-0.07, 0.09)	0.88	0.6 (0.45, 0.75)	7.5e-15	0.33 (-0.03, 0.69)	0.076
S	0.41 (0.3, 0.52)	8.3e-13	0.21 (0.02, 0.4)	0.03	0 (-0.07, 0.08)	0.91	0 (-0.1, 0.1)	0.98	0.35 (0.2, 0.5)	7.5e-6	0.19 (-0.12, 0.51)	0.23
S1	0.6 (0.49, 0.7)	7.9e-28	0.32 (0.12, 0.52)	0.0019	0.06 (-0.01, 0.14)	0.089	0.05 (-0.03, 0.13)	0.25	0.5 (0.35, 0.65)	9e-11	0.26 (-0.09, 0.6)	0.15
**All IL-2 producing cells**												
U	0.1 (0.02, 0.19)	0.013	0.14 (0.04, 0.24)	0.0073	-0.06 (-0.13, 0)	0.054	-0.08 (-0.15, -0.01)	0.023	0.2 (0.09, 0.31)	0.00028	0.26 (0.12, 0.4)	0.00028
CMV	0.19 (0.04, 0.34)	0.016	0.19 (-0.08, 0.46)	0.17	-0.1 (-0.21, 0.01)	0.073	-0.23 (-0.4, -0.05)	0.012	0.35 (0.14, 0.55)	0.00087	0.44 (0.04, 0.85)	0.03
M	1.39 (1.29, 1.5)	9.3e-119	1.53 (1.35, 1.71)	9.5e-57	0.21 (0.13, 0.28)	4.1e-7	0.2 (0.12, 0.29)	4.1e-6	1.16 (1.02, 1.3)	1.7e-52	1.29 (1.07, 1.51)	1.7e-27
N	1.42 (1.34, 1.51)	2e-185	1.63 (1.5, 1.77)	2.9e-104	0.15 (0.09, 0.21)	3.2e-6	0.19 (0.13, 0.26)	6.2e-9	1.27 (1.17, 1.37)	1.6e-96	1.43 (1.3, 1.56)	4e-82
S	1.04 (0.95, 1.13)	1.5e-97	1.16 (1.03, 1.28)	6.8e-66	0.13 (0.06, 0.2)	0.00012	0.16 (0.09, 0.23)	1.1e-5	0.88 (0.76, 0.99)	2.1e-43	0.92 (0.78, 1.07)	2e-31
S1	1.45 (1.37, 1.54)	6.7e-173	1.73 (1.57, 1.89)	1e-87	0.2 (0.13, 0.26)	2.3e-8	0.29 (0.21, 0.36)	1.1e-13	1.23 (1.12, 1.34)	4.6e-85	1.34 (1.21, 1.47)	1.5e-70
**IFN-γ single positive**												
U	0 (-0.01, 0.02)	0.58	0.11 (-0.21, 0.43)	0.5	0 (-0.01, 0.01)	0.92	-0.11 (-0.33, 0.11)	0.32	0 (-0.02, 0.01)	0.87	0 (-0.44, 0.45)	0.98
CMV	0.07 (-0.04, 0.17)	0.21	0.63 (0.09, 1.16)	0.023	-0.03 (-0.11, 0.05)	0.51	-0.14 (-0.43, 0.14)	0.33	0.07 (-0.07, 0.21)	0.32	0.44 (-0.21, 1.1)	0.18
M	0.04 (0.01, 0.06)	0.019	0.67 (0.39, 0.96)	3.6e-6	0.02 (0, 0.04)	0.1	0.13 (-0.04, 0.31)	0.14	0 (-0.03, 0.03)	0.82	0.44 (0.05, 0.82)	0.027

N	0.06 (0.04, 0.08)	1e-7	1.42 (1.14, 1.7)	2e-22	-0.01 (-0.03, 0.01)	0.56	-0.1 (-0.25, 0.05)	0.19	0.08 (0.05, 0.1)	7.3e-10	1.59 (1.25, 1.92)	3.6e-19
S	0.01 (-0.01, 0.02)	0.34	0.35 (0.11, 0.59)	0.0041	0 (-0.01, 0.01)	0.96	-0.08 (-0.24, 0.09)	0.37	0 (-0.02, 0.02)	0.78	0.32 (-0.02, 0.65)	0.062
S1	0.03 (0.01, 0.05)	0.00085	1.13 (0.86, 1.4)	6.6e-16	0.01 (-0.01, 0.02)	0.39	0.15 (0, 0.29)	0.048	0.03 (0.01, 0.05)	0.0059	0.96 (0.6, 1.32)	2.5e-7
**TNF-α single positive**												
U	0.1 (-0.03, 0.22)	0.13	-0.04 (-0.24, 0.17)	0.73	-0.12 (-0.21, -0.03)	0.013	-0.12 (-0.22, -0.02)	0.019	0.18 (0.01, 0.34)	0.033	0.08 (-0.26, 0.42)	0.66
CMV	0.08 (-0.02, 0.19)	0.13	-0.01 (-0.18, 0.16)	0.89	-0.06 (-0.14, 0.01)	0.11	-0.12 (-0.2, -0.03)	0.011	0.13 (-0.01, 0.27)	0.067	0.09 (-0.2, 0.38)	0.56
M	0.35 (0.24, 0.46)	2.3e-9	0.13 (-0.08, 0.34)	0.22	0.01 (-0.07, 0.09)	0.87	-0.01 (-0.11, 0.08)	0.81	0.31 (0.16, 0.47)	1e-4	0.15 (-0.21, 0.5)	0.41
N	0.43 (0.32, 0.54)	1.9e-13	0.13 (-0.1, 0.35)	0.27	-0.02 (-0.1, 0.06)	0.67	-0.05 (-0.14, 0.05)	0.33	0.42 (0.26, 0.57)	2.6e-7	0.18 (-0.21, 0.57)	0.36
S	0.25 (0.14, 0.37)	1.8e-5	0.07 (-0.14, 0.28)	0.53	-0.04 (-0.13, 0.04)	0.31	-0.05 (-0.15, 0.05)	0.36	0.24 (0.09, 0.4)	0.0022	0.1 (-0.26, 0.45)	0.59
S1	0.34 (0.22, 0.45)	1.1e-8	0.08 (-0.14, 0.3)	0.49	-0.01 (-0.09, 0.08)	0.9	-0.04 (-0.14, 0.06)	0.41	0.3 (0.14, 0.46)	0.00028	0.1 (-0.28, 0.48)	0.59
**IL-2 single positive**												
U	0 (-0.06, 0.06)	0.98	0.02 (-0.1, 0.14)	0.73	-0.03 (-0.07, 0.02)	0.22	-0.09 (-0.18, 0)	0.041	0.04 (-0.03, 0.12)	0.23	0.15 (-0.01, 0.32)	0.07
CMV	0.01 (-0.04, 0.07)	0.66	0.03 (-0.08, 0.15)	0.58	-0.03 (-0.07, 0.02)	0.24	-0.05 (-0.13, 0.03)	0.22	0.05 (-0.02, 0.13)	0.17	0.11 (-0.05, 0.28)	0.19
M	0.24 (0.18, 0.31)	5.9e-13	0.4 (0.28, 0.51)	1.2e-11	0.08 (0.02, 0.13)	0.004	0.08 (0, 0.16)	0.041	0.18 (0.1, 0.26)	1.4e-5	0.35 (0.18, 0.51)	3.3e-5
N	0.24 (0.18, 0.31)	3e-14	0.48 (0.36, 0.59)	3.5e-16	0.05 (0, 0.1)	0.077	0.05 (-0.02, 0.13)	0.18	0.21 (0.13, 0.28)	1.4e-7	0.46 (0.31, 0.61)	6.1e-9
S	0.15 (0.09, 0.21)	5.9e-7	0.26 (0.15, 0.37)	5.7e-6	0.03 (-0.02, 0.07)	0.27	0.03 (-0.04, 0.11)	0.41	0.15 (0.07, 0.22)	0.00018	0.23 (0.07, 0.39)	0.0056
S1	0.23 (0.17, 0.3)	4.5e-12	0.41 (0.3, 0.52)	1.8e-12	0.03 (-0.02, 0.08)	0.24	0.06 (-0.02, 0.14)	0.13	0.21 (0.13, 0.29)	5.3e-7	0.38 (0.23, 0.53)	1.4e-6
**IFN-γ and TNF-α double positive**												
U	0.06 (0.01, 0.1)	0.023	1 (0.31, 1.68)	0.0047	-0.01 (-0.05, 0.04)	0.78	-0.18 (-0.64, 0.28)	0.44	0.06 (0, 0.12)	0.052	1.26 (0.53, 1.99)	0.00077
CMV	0.18 (-0.05, 0.4)	0.13	0.47 (-0.07, 1.02)	0.091	-0.03 (-0.2, 0.14)	0.73	-0.31 (-0.65, 0.03)	0.074	0.3 (0, 0.6)	0.053	0.87 (0.12, 1.63)	0.023
M	0.27 (0.11, 0.42)	0.001	0.4 (-0.09, 0.88)	0.11	0.1 (-0.02, 0.23)	0.12	0.11 (-0.22, 0.44)	0.51	0.14 (-0.07, 0.36)	0.19	0.26 (-0.42, 0.93)	0.46
N	0.51 (0.44, 0.58)	2.5e-44	1.94 (1.56, 2.31)	2.8e-23	0.07 (0.01, 0.13)	0.021	0.12 (-0.06, 0.3)	0.19	0.44 (0.37, 0.5)	8.2e-37	1.74 (1.47, 2.01)	1.7e-33
S	0.22 (0.09, 0.35)	0.00074	0.5 (0.04, 0.97)	0.035	0.05 (-0.05, 0.16)	0.32	0.09 (-0.21, 0.39)	0.55	0.15 (-0.02, 0.33)	0.09	0.35 (-0.26, 0.96)	0.27
S1	0.39 (0.34, 0.45)	3.4e-39	2.38 (1.96, 2.8)	2.3e-27	0.1 (0.05, 0.15)	0.00038	0.33 (0.18, 0.47)	1.2e-5	0.3 (0.24, 0.36)	3.5e-23	1.96 (1.64, 2.28)	7.1e-30
**IFN-γ and IL-2 double positive**												
U	0 (0, 0)	0.63	0.64 (0.1, 1.18)	0.02	-	-	-0.38 (-0.74, -0.02)	0.038	-	-	0.97 (0.35, 1.58)	0.0022
CMV	0.01 (0, 0.02)	0.13	0.4 (0.03, 0.77)	0.035	0 (-0.01, 0.01)	0.73	-0.01 (-0.28, 0.26)	0.95	0.01 (0, 0.02)	0.1	0.51 (0.06, 0.97)	0.028
M	0.02 (0.01, 0.03)	0.0013	1.95 (1.48, 2.42)	6.7e-16	0.02 (0.01, 0.03)	0.0016	0.26 (0.06, 0.46)	0.0093	0.01 (0, 0.01)	0.051	1.64 (1.12, 2.17)	1.4e-9
N	0.01 (0, 0.02)	0.027	1.56 (1.19, 1.93)	2.5e-16	0.01 (0, 0.02)	0.0075	0.25 (0.09, 0.42)	0.0029	0.01 (0, 0.01)	0.058	1.36 (0.94, 1.77)	4.1e-10
S	0 (0, 0.01)	0.12	1.14 (0.73, 1.56)	7.6e-8	0.01 (0, 0.01)	0.0037	0.26 (0.05, 0.47)	0.017	0 (0, 0)	0.6	0.89 (0.35, 1.42)	0.0012
S1	0.02 (0.01, 0.03)	0.00029	2.09 (1.6, 2.58)	1.3e-16	0.01 (0, 0.02)	0.0032	0.31 (0.14, 0.48)	0.00046	0.01 (0, 0.01)	0.0018	1.73 (1.2, 2.26)	3.6e-10
**TNF-α and IL-2 double positive**												
U	0.09 (0.02, 0.16)	0.01	0.18 (0.05, 0.32)	0.008	-0.02 (-0.07, 0.04)	0.53	-0.06 (-0.16, 0.04)	0.27	0.12 (0.03, 0.22)	0.011	0.27 (0.08, 0.47)	0.0057
CMV	0.08 (-0.01, 0.17)	0.09	0.09 (-0.01, 0.2)	0.084	-0.07 (-0.14, -0.01)	0.029	-0.09 (-0.17, -0.02)	0.0099	0.17 (0.05, 0.29)	0.0053	0.2 (0.05, 0.35)	0.01
M	1.45 (1.35, 1.55)	3.2e-138	2.11 (1.9, 2.32)	2.2e-75	0.22 (0.14, 0.3)	2.5e-7	0.28 (0.19, 0.36)	3.4e-10	1.2 (1.09, 1.31)	1.1e-80	1.77 (1.58, 1.95)	5.9e-65
N	1.34 (1.25, 1.42)	1.2e-153	1.91 (1.73, 2.09)	4.3e-84	0.16 (0.09, 0.24)	9.2e-6	0.25 (0.18, 0.32)	7.8e-11	1.14 (1.05, 1.24)	6.5e-90	1.62 (1.46, 1.79)	6.4e-67
S	1.01 (0.92, 1.1)	1.5e-93	1.44 (1.3, 1.58)	4.3e-75	0.13 (0.06, 0.2)	0.00029	0.19 (0.12, 0.26)	5.1e-7	0.84 (0.74, 0.94)	3.5e-51	1.16 (1.01, 1.31)	3.3e-44
S1	1.38 (1.29, 1.47)	3.4e-147	1.9 (1.71, 2.09)	3.4e-75	0.21 (0.13, 0.28)	1.6e-7	0.32 (0.24, 0.4)	7.1e-14	1.15 (1.05, 1.25)	3e-84	1.47 (1.3, 1.63)	1.4e-58
**Polyfunctional IFN-γ, TNF-α and IL-2 triple positive**												
U	0.02 (0, 0.04)	0.12	0.4 (0.11, 0.69)	0.0062	0 (-0.02, 0.02)	0.95	-0.17 (-0.37, 0.02)	0.082	0.03 (0, 0.05)	0.035	0.67 (0.28, 1.05)	0.00075
CMV	0.18 (-0.01, 0.36)	0.064	0.21 (-0.1, 0.52)	0.19	-0.1 (-0.24, 0.04)	0.15	-0.25 (-0.45, -0.05)	0.015	0.35 (0.1, 0.6)	0.0066	0.49 (0.03, 0.94)	0.038
M	0.93 (0.81, 1.04)	2.1e-51	1.41 (1.11, 1.72)	2.2e-19	0.2 (0.1, 0.29)	6e-5	0.12 (-0.01, 0.25)	0.06	0.7 (0.56, 0.85)	5.4e-21	1.26 (0.82, 1.7)	3e-8

N	0.87 (0.79, 0.94)	1.1e-92	2.71 (2.43, 2.99)	4.3e-71	0.11 (0.04, 0.18)	0.0021	0.13 (0.05, 0.22)	0.0016	0.77 (0.7, 0.84)	5.8e-76	2.59 (2.35, 2.83)	4.8e-76
S	0.58 (0.49, 0.66)	2.5e-39	1.45 (1.19, 1.72)	1.3e-25	0.12 (0.05, 0.19)	0.00099	0.16 (0.04, 0.28)	0.007	0.43 (0.33, 0.52)	1.8e-17	1.19 (0.81, 1.56)	7.5e-10
S1	0.99 (0.91, 1.07)	1.3e-103	3.26 (2.88, 3.65)	2.9e-56	0.22 (0.15, 0.3)	3.5e-9	0.32 (0.23, 0.41)	5.7e-12	0.74 (0.67, 0.81)	3.4e-72	2.8 (2.51, 3.09)	1.3e-64

We note that when unstimulated, all IFN-γ^+ and IL-2^+ as well as double positive IFN-γ^+TNF-α^+, and TNF-α^+IL-2^+ CD4^+ T-cell responses among cases were nominally stronger than that of controls, but the magnitude of these responses were much smaller than the difference between stimulated cells from cases and controls. For example, we detected 8% (95% CI: 1-15%) more IFN-γ^+TNF-α^+ CD4^+ T-cells in the unstimulated culture in cases than controls, while for cells responding to stimulation with S1, the difference was 322% (95% CI: 286-362%).

Supplementary Table 3. Effect of SARS-CoV-2 infection status and severity of COVID-19 on SARS-CoV-2 and CMV reactive CD8⁺ T-cell responses based on GLS and GLM models. Effects represent differences in the logarithm of the fraction (GLS) and absolute count (GLM-quasi-Poisson) of stimulated cells between cases (n=764) and controls (n=387), per unit of the severity scale in cases (n=543), and between cases with the mildest disease severity (n=207) and controls (n=387). P value corrected for multiple testing P<0.05/60=8.3E-04.

	Infection association (N cases=764, N controls=387)				Severity scale association (N=543)				Mild infection association (N cases=207, N controls=387)			
	GLS		GLM – quasi-Poisson		GLS		GLM – quasi-Poisson		GLS		GLM – quasi-Poisson	
Protein	Effect (95% CI)	P	Effect (95% CI)	P	Effect (95% CI)	P	Effect (95% CI)	P	Effect (95% CI)	P	Effect (95% CI)	P
**All IFN-γ producing cells**												
U	0 (-0.1, 0.09)	0.93	0.67 (-0.56, 1.9)	0.28	-0.07 (-0.14, 0.01)	0.08	0.27 (-0.59, 1.12)	0.54	0.02 (-0.11, 0.16)	0.74	0.45 (-0.17, 1.07)	0.15
CMV	0.15 (-0.13, 0.43)	0.29	0.02 (-0.15, 0.19)	0.83	-0.02 (-0.23, 0.19)	0.85	0 (-0.12, 0.12)	1	0.24 (-0.14, 0.61)	0.21	0.03 (-0.23, 0.3)	0.81
M	0.21 (0.12, 0.31)	1.3e-05	0.8 (-0.02, 1.62)	0.055	0.04 (-0.03, 0.12)	0.26	0 (-0.58, 0.59)	0.99	0.13 (0, 0.25)	0.046	0.19 (-0.32, 0.7)	0.47
N	1.07 (0.94, 1.21)	1.7e-50	1.59 (1.27, 1.91)	1.5e-21	0.08 (-0.03, 0.19)	0.17	0.02 (-0.1, 0.14)	0.71	0.89 (0.74, 1.04)	1.1e-28	1.45 (1.08, 1.81)	3.5e-14
S	0.22 (0.11, 0.32)	1e-04	0.35 (-0.06, 0.77)	0.098	-0.03 (-0.12, 0.05)	0.45	-0.02 (-0.27, 0.24)	0.91	0.22 (0.08, 0.36)	0.0022	0.3 (-0.27, 0.88)	0.3
S1	0.48 (0.36, 0.6)	8.5e-15	0.49 (0.02, 0.97)	0.042	-0.04 (-0.13, 0.06)	0.46	-0.04 (-0.21, 0.12)	0.6	0.45 (0.29, 0.61)	3.6e-08	0.47 (-0.26, 1.21)	0.21
**All TNF-α producing cells**												
U	-0.01 (-0.16, 0.14)	0.92	-0.03 (-0.25, 0.19)	0.76	-0.07 (-0.19, 0.04)	0.2	-0.05 (-0.22, 0.13)	0.59	0.03 (-0.17, 0.23)	0.79	0.04 (-0.27, 0.35)	0.79
CMV	0.05 (-0.14, 0.25)	0.59	0.03 (-0.15, 0.2)	0.75	-0.03 (-0.17, 0.12)	0.72	0 (-0.12, 0.13)	0.94	0.12 (-0.14, 0.38)	0.36	0.05 (-0.22, 0.31)	0.72
M	0.05 (-0.1, 0.2)	0.53	-0.11 (-0.4, 0.18)	0.46	-0.05 (-0.16, 0.05)	0.33	-0.07 (-0.21, 0.07)	0.35	0.04 (-0.16, 0.24)	0.69	-0.09 (-0.59, 0.42)	0.74
N	0.3 (0.16, 0.45)	2.7e-05	0.05 (-0.14, 0.23)	0.62	0.02 (-0.08, 0.12)	0.74	-0.06 (-0.16, 0.04)	0.26	0.21 (0.01, 0.41)	0.036	0.07 (-0.21, 0.36)	0.62
S	0.07 (-0.08, 0.22)	0.35	-0.01 (-0.2, 0.18)	0.92	-0.09 (-0.2, 0.03)	0.13	-0.04 (-0.16, 0.08)	0.52	0.07 (-0.13, 0.27)	0.5	-0.02 (-0.29, 0.25)	0.87
S1	0.1 (-0.04, 0.25)	0.16	0 (-0.18, 0.18)	0.99	-0.05 (-0.16, 0.05)	0.34	-0.07 (-0.18, 0.04)	0.23	0.07 (-0.13, 0.26)	0.5	0.02 (-0.26, 0.29)	0.91
**All IL-2 producing cells**												
U	-0.09 (-0.25, 0.07)	0.27	-0.09 (-0.3, 0.12)	0.41	-0.04 (-0.16, 0.08)	0.55	0.09 (-0.07, 0.26)	0.27	-0.04 (-0.25, 0.16)	0.7	-0.17 (-0.47, 0.13)	0.26
CMV	-0.01 (-0.15, 0.13)	0.91	-0.01 (-0.19, 0.17)	0.89	0.03 (-0.08, 0.13)	0.62	0.05 (-0.09, 0.19)	0.47	-0.04 (-0.22, 0.15)	0.7	-0.03 (-0.29, 0.22)	0.8
M	0.01 (-0.15, 0.17)	0.87	-0.03 (-0.26, 0.19)	0.76	0.03 (-0.09, 0.15)	0.6	0.07 (-0.1, 0.24)	0.42	0.01 (-0.2, 0.22)	0.92	-0.1 (-0.43, 0.24)	0.57
N	0.09 (-0.06, 0.24)	0.23	-0.02 (-0.25, 0.21)	0.86	0.05 (-0.06, 0.16)	0.41	0.06 (-0.1, 0.22)	0.46	0.05 (-0.15, 0.26)	0.61	-0.08 (-0.43, 0.26)	0.63
S	0 (-0.16, 0.15)	0.98	0.01 (-0.25, 0.26)	0.96	-0.03 (-0.14, 0.09)	0.67	0.11 (-0.09, 0.3)	0.28	0.01 (-0.19, 0.22)	0.91	-0.06 (-0.41, 0.28)	0.72
S1	0.02 (-0.13, 0.18)	0.77	-0.07 (-0.3, 0.16)	0.57	0.01 (-0.11, 0.12)	0.89	0.06 (-0.1, 0.22)	0.47	0 (-0.21, 0.2)	0.98	-0.15 (-0.5, 0.19)	0.39
**IFN-γ single positive**												
U	-0.02 (-0.04, 0)	0.074	-0.24 (-0.71, 0.23)	0.31	0 (-0.02, 0.01)	0.58	0.01 (-0.32, 0.35)	0.94	-0.02 (-0.06, 0.01)	0.19	-0.25 (-0.95, 0.44)	0.48
CMV	0.05 (-0.11, 0.21)	0.54	0.19 (-0.13, 0.51)	0.23	-0.02 (-0.14, 0.11)	0.79	0.08 (-0.15, 0.31)	0.48	0.04 (-0.17, 0.25)	0.72	0.11 (-0.31, 0.53)	0.62
M	0.05 (0.01, 0.08)	0.004	0.51 (0.12, 0.91)	0.011	0.03 (0, 0.05)	0.02	0.33 (0.12, 0.55)	0.0027	0.01 (-0.03, 0.05)	0.57	0.14 (-0.52, 0.8)	0.68
N	0.4 (0.33, 0.48)	2.1e-23	2.13 (1.65, 2.6)	6.5e-18	0.02 (-0.05, 0.08)	0.65	-0.01 (-0.16, 0.14)	0.94	0.34 (0.26, 0.41)	1.8e-19	2.02 (1.6, 2.44)	5.9e-20
S	0.03 (-0.01, 0.06)	0.11	0.05 (-0.66, 0.76)	0.89	0 (-0.02, 0.03)	0.95	0.06 (-0.18, 0.29)	0.64	0.03 (-0.02, 0.08)	0.24	-0.06 (-1.39, 1.28)	0.94
S1	0.09 (0.05, 0.13)	3.3e-06	1.24 (0.86, 1.62)	2.6e-10	0.01 (-0.03, 0.04)	0.76	0.16 (-0.03, 0.35)	0.11	0.1 (0.05, 0.14)	3.2e-05	1.17 (0.71, 1.63)	9.3e-07
**TNF-α single positive**												

U	0.01 (-0.14, 0.16)	0.91	-0.06 (-0.26, 0.14)	0.56	-0.08 (-0.19, 0.03)	0.13	-0.13 (-0.26, 0)	0.059	0.07 (-0.13, 0.26)	0.5	0.08 (-0.23, 0.4)	0.6
CMV	0.05 (-0.11, 0.2)	0.55	0.1 (-0.21, 0.41)	0.54	-0.02 (-0.14, 0.09)	0.67	0.03 (-0.21, 0.26)	0.83	0.1 (-0.11, 0.3)	0.35	0.13 (-0.31, 0.58)	0.56
M	0.02 (-0.12, 0.17)	0.74	-0.2 (-0.55, 0.15)	0.27	-0.08 (-0.19, 0.02)	0.12	-0.09 (-0.21, 0.02)	0.11	0.05 (-0.14, 0.25)	0.58	-0.1 (-0.7, 0.51)	0.75
N	0.06 (-0.08, 0.2)	0.41	-0.07 (-0.26, 0.12)	0.46	-0.04 (-0.15, 0.07)	0.46	-0.1 (-0.21, 0.02)	0.095	0.03 (-0.16, 0.23)	0.73	-0.01 (-0.3, 0.28)	0.96
S	0.04 (-0.11, 0.19)	0.59	-0.03 (-0.21, 0.15)	0.75	-0.1 (-0.21, 0.01)	0.084	-0.08 (-0.19, 0.04)	0.2	0.06 (-0.13, 0.25)	0.55	-0.03 (-0.29, 0.24)	0.85
S1	0.02 (-0.12, 0.17)	0.75	-0.02 (-0.19, 0.16)	0.87	-0.07 (-0.18, 0.04)	0.22	-0.08 (-0.2, 0.04)	0.19	0.01 (-0.19, 0.2)	0.96	0.02 (-0.25, 0.28)	0.89
**IL-2 single positive**												
U	-0.12 (-0.28, 0.04)	0.15	-0.07 (-0.29, 0.14)	0.51	-0.03 (-0.15, 0.09)	0.63	0.13 (-0.05, 0.3)	0.17	-0.06 (-0.27, 0.15)	0.58	-0.18 (-0.47, 0.1)	0.21
CMV	-0.11 (-0.27, 0.05)	0.16	-0.02 (-0.26, 0.21)	0.84	0.03 (-0.09, 0.16)	0.61	0.16 (-0.03, 0.36)	0.092	-0.14 (-0.35, 0.07)	0.18	-0.16 (-0.47, 0.15)	0.32
M	-0.02 (-0.18, 0.14)	0.78	-0.01 (-0.24, 0.21)	0.91	0.03 (-0.09, 0.15)	0.58	0.1 (-0.08, 0.28)	0.27	-0.02 (-0.23, 0.19)	0.84	-0.09 (-0.4, 0.22)	0.57
N	-0.07 (-0.23, 0.09)	0.39	-0.03 (-0.26, 0.2)	0.81	0.03 (-0.09, 0.15)	0.65	0.09 (-0.09, 0.27)	0.32	-0.09 (-0.3, 0.12)	0.41	-0.12 (-0.44, 0.21)	0.47
S	-0.02 (-0.18, 0.13)	0.77	0.03 (-0.23, 0.28)	0.83	-0.03 (-0.14, 0.09)	0.66	0.13 (-0.07, 0.33)	0.21	0.01 (-0.2, 0.21)	0.96	-0.05 (-0.38, 0.27)	0.75
S1	-0.01 (-0.16, 0.15)	0.95	-0.05 (-0.28, 0.18)	0.68	0 (-0.12, 0.12)	0.97	0.09 (-0.08, 0.27)	0.29	-0.01 (-0.22, 0.2)	0.95	-0.15 (-0.47, 0.17)	0.36
**IFN-γ and TNF-α double positive**												
U	0.01 (-0.08, 0.1)	0.8	0.84 (-0.58, 2.26)	0.25	-0.05 (-0.12, 0.02)	0.15	0.29 (-0.62, 1.2)	0.53	0.05 (-0.07, 0.18)	0.41	0.58 (-0.12, 1.28)	0.1
CMV	0.13 (-0.15, 0.42)	0.36	0.01 (-0.16, 0.19)	0.9	-0.03 (-0.25, 0.18)	0.77	0 (-0.12, 0.12)	0.95	0.24 (-0.14, 0.62)	0.22	0.02 (-0.25, 0.29)	0.88
M	0.16 (0.07, 0.24)	0.00029	0.92 (-0.08, 1.92)	0.072	0.03 (-0.03, 0.1)	0.33	-0.03 (-0.7, 0.64)	0.93	0.1 (-0.01, 0.21)	0.071	0.2 (-0.42, 0.83)	0.52
N	0.81 (0.69, 0.93)	4.4e-38	1.44 (1.06, 1.81)	1.1e-13	0.07 (-0.03, 0.17)	0.18	0.03 (-0.1, 0.17)	0.63	0.65 (0.52, 0.79)	3.6e-20	1.28 (0.8, 1.75)	1.7e-07
S	0.18 (0.08, 0.27)	0.00033	0.42 (-0.08, 0.92)	0.096	-0.01 (-0.09, 0.07)	0.74	0.01 (-0.3, 0.33)	0.93	0.16 (0.04, 0.29)	0.0099	0.32 (-0.31, 0.96)	0.32
S1	0.41 (0.29, 0.52)	4.7e-12	0.46 (-0.09, 1.01)	0.1	-0.03 (-0.13, 0.06)	0.45	-0.08 (-0.27, 0.11)	0.4	0.38 (0.23, 0.53)	8.2e-07	0.47 (-0.38, 1.31)	0.28
**IFN-γ and IL-2 double positive**												
U	0 (-0.03, 0.03)	0.96	-0.2 (-0.57, 0.17)	0.29	0 (-0.02, 0.02)	0.99	0.03 (-0.26, 0.33)	0.83	-0.02 (-0.06, 0.01)	0.22	-0.44 (-1.02, 0.13)	0.13
CMV	0.01 (-0.03, 0.05)	0.59	0.03 (-0.36, 0.41)	0.88	0 (-0.03, 0.03)	0.99	0.02 (-0.19, 0.22)	0.88	0.02 (-0.03, 0.07)	0.48	0.03 (-0.34, 0.4)	0.88
M	-0.01 (-0.05, 0.02)	0.49	-0.12 (-0.46, 0.21)	0.47	-0.01 (-0.03, 0.01)	0.34	-0.09 (-0.36, 0.17)	0.5	-0.02 (-0.06, 0.02)	0.37	-0.14 (-0.6, 0.31)	0.54
N	0.09 (0.05, 0.14)	9.2e-06	0.66 (0.35, 0.96)	2.6e-05	0.02 (-0.02, 0.05)	0.35	0.04 (-0.15, 0.23)	0.66	0.05 (0.01, 0.1)	0.011	0.47 (0.07, 0.87)	0.021
S	0 (-0.04, 0.03)	0.97	-0.12 (-0.44, 0.2)	0.45	-0.01 (-0.04, 0.02)	0.52	-0.06 (-0.31, 0.19)	0.63	0 (-0.05, 0.04)	0.98	-0.1 (-0.54, 0.33)	0.64
S1	-0.02 (-0.05, 0.01)	0.22	-0.24 (-0.55, 0.08)	0.14	-0.01 (-0.03, 0.01)	0.48	0.07 (-0.18, 0.32)	0.6	-0.03 (-0.08, 0.02)	0.24	-0.51 (-1.05, 0.03)	0.063
**TNF-α and IL-2 double positive**												
U	0 (-0.17, 0.17)	0.99	-0.13 (-0.4, 0.13)	0.32	-0.03 (-0.15, 0.1)	0.69	0 (-0.16, 0.16)	0.99	0.01 (-0.22, 0.23)	0.96	-0.14 (-0.59, 0.3)	0.52
CMV	0.03 (-0.13, 0.2)	0.7	-0.13 (-0.42, 0.15)	0.37	0 (-0.12, 0.13)	0.97	0.02 (-0.15, 0.19)	0.82	-0.01 (-0.23, 0.21)	0.91	-0.15 (-0.63, 0.32)	0.52
M	0.03 (-0.12, 0.19)	0.67	-0.11 (-0.39, 0.18)	0.47	0.01 (-0.1, 0.13)	0.83	-0.02 (-0.19, 0.15)	0.82	0.01 (-0.2, 0.21)	0.96	-0.12 (-0.59, 0.36)	0.63
N	0.11 (-0.05, 0.27)	0.19	-0.12 (-0.42, 0.18)	0.43	0.03 (-0.1, 0.15)	0.69	-0.02 (-0.18, 0.14)	0.8	0.07 (-0.14, 0.29)	0.51	-0.1 (-0.61, 0.4)	0.7
S	0.02 (-0.14, 0.18)	0.78	-0.06 (-0.38, 0.27)	0.72	0.01 (-0.11, 0.13)	0.9	0.05 (-0.15, 0.25)	0.61	-0.03 (-0.24, 0.18)	0.77	-0.11 (-0.61, 0.39)	0.67
S1	0.05 (-0.11, 0.21)	0.51	-0.13 (-0.44, 0.18)	0.41	0.02 (-0.1, 0.14)	0.72	-0.03 (-0.21, 0.15)	0.76	0.01 (-0.2, 0.22)	0.94	-0.18 (-0.71, 0.36)	0.52
**Polyfunctional IFN-γ, TNF-α and IL-2 triple positive**												
U	0.14 (-0.04, 0.33)	0.13	0.08 (-0.13, 0.29)	0.44	-0.03 (-0.17, 0.11)	0.66	-0.12 (-0.26, 0.03)	0.11	0.15 (-0.09, 0.4)	0.22	0.22 (-0.09, 0.54)	0.16
CMV	0.03 (0.01, 0.04)	0.00042	1.9 (1.05, 2.76)	1.3e-05	0.01 (0, 0.02)	0.15	0.26 (-0.02, 0.54)	0.074	0.01 (0, 0.02)	0.17	1.46 (0.27, 2.64)	0.016
M	0.3 (0.24, 0.37)	5.6e-20	3.26 (2.45, 4.07)	5.2e-15	0.02 (-0.03, 0.08)	0.4	0 (-0.18, 0.19)	0.99	0.26 (0.21, 0.32)	1.1e-19	3.21 (2.53, 3.89)	2.2e-19
N	0.04 (0.02, 0.06)	0.0012	1.1 (0.41, 1.78)	0.0017	-0.02 (-0.04, 0)	0.053	-0.43 (-0.85, -0.01)	0.045	0.06 (0.03, 0.09)	0.00025	1.48 (0.67, 2.29)	0.00039
S	0.05 (0.02, 0.08)	0.00043	0.91 (0.38, 1.43)	0.00073	-0.01 (-0.03, 0.02)	0.6	0.03 (-0.22, 0.28)	0.81	0.05 (0.02, 0.09)	0.0048	0.83 (0.09, 1.56)	0.028
S1	0.14 (-0.04, 0.33)	0.13	0.08 (-0.13, 0.29)	0.44	-0.03 (-0.17, 0.11)	0.66	-0.12 (-0.26, 0.03)	0.11	0.15 (-0.09, 0.4)	0.22	0.22 (-0.09, 0.54)	0.16

Supplementary Table 4 Stepwise conditional analysis of HLA alleles associating with T cell responses. Analysis performed on 742 HLA genotyped SARS-CoV-2 cases.

HLA allele associations with N reactive CD8+ T-cell responses

HLA	Allele frequency	P value	Effect (95% CI)	P value$_{adj}$	Effect adj (95% CI)	Covariate
HLA-B*07:02	18.3%	7.9e-46	1.08 (0.93, 1.22)	-	-	-
HLA-C*07:02	20.6%	1.0e-37	0.94 (0.80, 1.08.)	0.94	0.01 (-0.30, 0.33)	HLA-B*07:02
HLA-DRB1*15:01	22.3%	1.4e-13	0.54 (0.40. 0.69)	0.81	-0.02 (-0.17, 0.13)	HLA-B*07:02
HLA-DQB1*06:02	21.6%	6.4e-13	0.54 (0.39, 0.69)	0.99	0.00 (-0.19, 0.19)	HLA-B*07:02
HLA-DQA1*01:02	30.6%	1.3e-9	0.40 (0.27, 0.53)	0.70	-0.03 (-0.15, 0.10)	HLA-B*07:02

HLA allele associations with S1 reactive CD8+ T-cell responses

HLA	Allele frequency	P value	Effect	P value $_{adj}$	Effect $_{adj}$	Covariate
HLA-C*07:02	20.6%	3.0e-12	0.46 (0.33, 0.58)	-	-	-
HLA-B*07:02	18.3%	6.2e-12	0.47 (0.33, 0.59)	0.21	0.20 (-0.11, 0.52)	HLA-C*07:02
HLA-A*01:01	10.7%	3.8e-9	-0.49 (-0.65, -0.33)	1.5E-7	-0.42 (-0.58, -0.27)	HLA-C*07:02

HLA allele associations with Sreactive CD8+ T-cell responses

HLA	Allele frequency	P value	Effect	P value$_{adj}$	Effect $_{adj}$	Covariate 1
HLA-G*01:04	10.9%	7.5e-14	0.51 (0.38, 0.65)	-	-	-
HLA-A*24:02	7.5%	2.1e-08	0.48 (0.31, 0.65)	0.55	0.07 (-0.16, 0.30)	HLA-G*01:04

HLA allele associations with CMV reactive CD8+ T-cell responses

HLA allele	Allele frequency	P value	Effect	Pvalue$_{adj}$	Effect$_{adj}$	Covariate
HLA-C*07:02	20.6%	3.8e-8	0.82 (0.53, 1.11)	-	-	-
HLA-B*07:02	18.3%	5.8e-8	0.84 (0.54, 1.14)	0.32	0.37 (-0.36, 1.10)	HLA-C*07:02

Supplementary Table 5. Association of CD8⁺ and CD4⁺ T-cell response associating HLA alleles with severity score (n=1643). HLA severity score analaysis were done on all Icelanders that had severity score data available at the time of the analysis.

Association of CD8+ T-cell response associating HLA-alleles with severity score

Protein	HLA allele	AF	P value	Effect
N	HLA-B*07:02	18.26	0.127	0.088
S1	HLA-C*07:02	20.62	0.692	0.019
S1	HLA-A*01:01	10.72	0.044	0.122
S	HLA-G*01:04	10.98	0.805	0.014
Association of CD4+ T-cell response associating HLA-alleles with severity score				
M	DQA1*05:05	5.945	0.392	-0.061

Supplementary Table 6. Relationship between sex and SARS-CoV-2 reactive $CD4^+$ and $CD8^+$ T-cell responses in SARS-CoV-2 cases and uninfected controls. Correlations are shown for all stimulation conditions: unstimulated, positive control CMV, and the SARS-CoV-2 protein stimulation (M, N, S and S1 subunit). Effect represents differences in the logarithm of the fraction of cytokine secreting cells in males ($CD4^+$ n cases=356, n controls=157; $CD8^+$ n cases=355, n controls=156) vs females ($CD4^+$ n cases=411, n controls=235; $CD8^+$ n cases=409, n controls=231).

		Unstimulated		CMV		M		N		S		S1	
**Case status**	**Cell**	**Effect (95% CI)**	**P**	**Effect (95% CI)**	**P**	**Effect (95% CI)**	**P**	**Effect (95% CI)**	**P**	**Effect (95% CI)**	**P**	**Effect (95% CI)**	**P**
$CD4^+$ T-cells													
Case	IFN+TNF+IL2+	0 (-0.02, 0.03)	0.87	-0.02 (-0.29, 0.25)	0.89	-0.07 (-0.21, 0.06)	0.28	0 (-0.03, 0.03)	0.89	-0.07 (-0.15, 0.02)	0.14	-0.01 (-0.03, 0.02)	0.55
Control	IFN+TNF+IL2+	0 (-0.03, 0.02)	0.75	0.01 (-0.2, 0.22)	0.93	-0.05 (-0.2, 0.1)	0.5	-0.11 (-0.21, 0)	0.05	-0.01 (-0.12, 0.1)	0.88	-0.12 (-0.23, 0)	0.057
Case	IFN+TNF+IL2-	0 (-0.05, 0.05)	0.92	0.03 (-0.3, 0.35)	0.88	-0.12 (-0.36, 0.11)	0.31	0 (-0.04, 0.05)	0.83	-0.15 (-0.34, 0.03)	0.11	0.03 (-0.01, 0.06)	0.11
Control	IFN+TNF+IL2-	0.01 (-0.05, 0.08)	0.71	0.03 (-0.23, 0.29)	0.83	-0.02 (-0.2, 0.17)	0.86	-0.05 (-0.15, 0.04)	0.27	0.01 (-0.14, 0.17)	0.86	-0.05 (-0.14, 0.03)	0.21
Case	IFN-TNF+IL2+	-0.08 (-0.18, 0.01)	0.092	-0.01 (-0.14, 0.13)	0.93	-0.04 (-0.14, 0.05)	0.37	-0.06 (-0.15, 0.03)	0.18	-0.09 (-0.19, 0.01)	0.069	-0.01 (-0.11, 0.09)	0.82
Control	IFN-TNF+IL2+	-0.05 (-0.13, 0.04)	0.26	0.06 (-0.04, 0.16)	0.22	-0.14 (-0.27, -0.01)	0.033	-0.09 (-0.2, 0.03)	0.13	-0.11 (-0.22, 0)	0.056	-0.17 (-0.29, -0.05)	0.0051
Case	IFN+	-0.15 (-0.26, -0.03)	0.014	-0.06 (-0.29, 0.16)	0.59	-0.09 (-0.24, 0.06)	0.25	-0.01 (-0.12, 0.1)	0.89	-0.13 (-0.25, 0)	0.058	-0.04 (-0.16, 0.08)	0.49
Control	IFN+	-0.04 (-0.14, 0.05)	0.38	0 (-0.17, 0.17)	0.98	-0.1 (-0.22, 0.03)	0.12	-0.07 (-0.17, 0.02)	0.14	-0.09 (-0.19, 0.02)	0.1	-0.14 (-0.24, -0.03)	0.012
Case	IL2+	-0.02 (-0.09, 0.05)	0.54	0.02 (-0.31, 0.35)	0.92	-0.14 (-0.39, 0.11)	0.28	0.03 (-0.04, 0.1)	0.47	-0.15 (-0.34, 0.05)	0.15	0.02 (-0.03, 0.07)	0.41
Control	IL2+	0.04 (-0.04, 0.12)	0.32	0.04 (-0.21, 0.3)	0.74	-0.07 (-0.25, 0.12)	0.48	-0.09 (-0.2, 0.03)	0.13	-0.02 (-0.18, 0.13)	0.78	-0.11 (-0.23, 0.01)	0.082
Case	TNF+	0.09 (-0.08, 0.26)	0.29	0.02 (-0.16, 0.2)	0.85	0.1 (-0.08, 0.29)	0.27	0.16 (0, 0.33)	0.056	0.1 (-0.07, 0.27)	0.24	0.09 (-0.08, 0.25)	0.33
Control	TNF+	-0.01 (-0.14, 0.13)	0.89	-0.05 (-0.19, 0.09)	0.49	-0.09 (-0.21, 0.03)	0.14	-0.06 (-0.16, 0.04)	0.24	-0.06 (-0.18, 0.06)	0.31	-0.05 (-0.16, 0.06)	0.36
$CD8^+$ T-cells													
Case	IFN+TNF+IL2+	0 (-0.02, 0.03)	0.97	-0.09 (-0.36, 0.18)	0.5	0 (-0.01, 0.02)	0.68	-0.01 (-0.02, 0.01)	0.5	0.01 (-0.02, 0.03)	0.62	-0.01 (-0.05, 0.02)	0.47
Control	IFN+TNF+IL2+	0 (-0.01, 0.02)	0.59	-0.22 (-0.43, -0.01)	0.04	-0.01 (-0.03, 0.01)	0.4	-0.05 (-0.15, 0.04)	0.24	-0.02 (-0.05, 0.01)	0.24	0 (-0.04, 0.04)	0.97

Case	IFN+TNF+IL2-	-0.13 (-0.26, 0)	0.047	-0.13 (-0.54, 0.28)	0.53	-0.07 (-0.19, 0.04)	0.23	-0.06 (-0.17, 0.04)	0.25	-0.04 (-0.16, 0.08)	0.52	-0.05 (-0.2, 0.1)	0.51
Control	IFN+TNF+IL2-	0 (-0.11, 0.1)	0.94	-0.17 (-0.49, 0.15)	0.3	-0.03 (-0.14, 0.08)	0.57	-0.13 (-0.28, 0.03)	0.11	-0.08 (-0.2, 0.04)	0.17	-0.05 (-0.2, 0.09)	0.46
Case	IFN-TNF+IL2+	-0.21 (-0.46, 0.04)	0.1	-0.05 (-0.29, 0.2)	0.7	-0.01 (-0.24, 0.22)	0.93	-0.03 (-0.27, 0.21)	0.81	-0.08 (-0.32, 0.15)	0.48	0 (-0.23, 0.24)	0.98
Control	IFN-TNF+IL2+	-0.2 (-0.39, -0.01)	0.039	-0.28 (-0.46, -0.09)	0.0042	-0.22 (-0.4, -0.04)	0.016	-0.18 (-0.37, 0.01)	0.059	-0.22 (-0.4, -0.03)	0.021	-0.12 (-0.3, 0.06)	0.19
Case	IFN+	-0.33 (-0.56, -0.1)	0.0049	-0.2 (-0.4, 0)	0.048	-0.15 (-0.37, 0.08)	0.21	-0.17 (-0.4, 0.05)	0.14	-0.16 (-0.39, 0.07)	0.16	-0.18 (-0.41, 0.05)	0.12
Control	IFN+	-0.24 (-0.42, -0.06)	0.0085	-0.28 (-0.43, -0.12)	0.00052	-0.23 (-0.41, -0.05)	0.012	-0.2 (-0.37, -0.03)	0.024	-0.25 (-0.42, -0.07)	0.0064	-0.17 (-0.35, 0)	0.054
Case	IL2+	-0.12 (-0.26, 0.02)	0.091	-0.1 (-0.51, 0.3)	0.61	-0.11 (-0.24, 0.02)	0.1	-0.09 (-0.21, 0.04)	0.17	-0.05 (-0.19, 0.09)	0.5	-0.02 (-0.18, 0.14)	0.79
Control	IL2+	0.01 (-0.1, 0.12)	0.86	-0.14 (-0.46, 0.17)	0.37	-0.02 (-0.14, 0.09)	0.69	-0.1 (-0.27, 0.07)	0.26	-0.03 (-0.17, 0.1)	0.61	-0.05 (-0.2, 0.1)	0.51
Case	TNF+	-0.11 (-0.33, 0.11)	0.33	-0.16 (-0.44, 0.12)	0.27	0.04 (-0.18, 0.27)	0.69	0.03 (-0.19, 0.25)	0.79	0.01 (-0.2, 0.23)	0.91	0.01 (-0.2, 0.22)	0.93
Control	TNF+	-0.16 (-0.33, 0)	0.057	-0.23 (-0.45, 0)	0.045	-0.18 (-0.34, -0.01)	0.033	-0.14 (-0.29, 0.01)	0.062	-0.17 (-0.33, 0)	0.05	-0.08 (-0.24, 0.08)	0.32
