# Supplementary material for: HLA alleles, disease severity, and age associate with T-cell responses following infection with SARS-CoV-2
Source: Commun Biol. 2022 Sep 6;5:914. doi: 10.1038/s42003-022-03893-w (PMC9446630; doi:10.1038/s42003-022-03893-w)
Supplement: Supplementary file 3 — Description of Additional Supplementary Files [file 42003_2022_3893_MOESM3_ESM.pdf]

Description of Additional Supplementary Files

File name: Supplementary Data 1
Description: List of SARS-CoV-2 peptides and their predicted binding across HLA alleles that associated with N-reactive CD8+ T cell responses.

File name: Supplementary Data 2
Description: Association of comorbidities with T-cell response.

File name: Supplementary Data 3
Description: Source data behind the graphs in the paper.
