# Supplementary material for: HLA alleles, disease severity, and age associate with T-cell responses following infection with SARS-CoV-2
Source: Commun Biol. 2022 Sep 6;5:914. doi: 10.1038/s42003-022-03893-w (PMC9446630; doi:10.1038/s42003-022-03893-w)
Supplement: Supplementary file 7 — Reporting summary [file 42003_2022_3893_MOESM7_ESM.pdf]

nature portfolio

Corresponding author(s): Thorunn Asta Olafsdottir and Kari Stefansson

Last updated by author(s): Jul 25, 2022

Reporting Summary

Nature Portfolio wishes to improve the reproducibility of the work that we publish. This form provides structure for consistency and transparency in reporting. For further information on Nature Portfolio policies, see our Editorial Policies and the Editorial Policy Checklist.

Statistics

For all statistical analyses, confirm that the following items are present in the figure legend, table legend, main text, or Methods section.

n/a	Confirmed	
☐	☒	The exact sample size (*n*) for each experimental group/condition, given as a discrete number and unit of measurement
☐	☒	A statement on whether measurements were taken from distinct samples or whether the same sample was measured repeatedly
☐	☒	The statistical test(s) used AND whether they are one- or two-sided<br>*Only common tests should be described solely by name; describe more complex techniques in the Methods section.*
☐	☒	A description of all covariates tested
☐	☒	A description of any assumptions or corrections, such as tests of normality and adjustment for multiple comparisons
☐	☒	A full description of the statistical parameters including central tendency (e.g. means) or other basic estimates (e.g. regression coefficient) AND variation (e.g. standard deviation) or associated estimates of uncertainty (e.g. confidence intervals)
☐	☒	For null hypothesis testing, the test statistic (e.g. *F*, *t*, *r*) with confidence intervals, effect sizes, degrees of freedom and *P* value noted<br>*Give P values as exact values whenever suitable.*
☒	☐	For Bayesian analysis, information on the choice of priors and Markov chain Monte Carlo settings
☒	☐	For hierarchical and complex designs, identification of the appropriate level for tests and full reporting of outcomes
☐	☒	Estimates of effect sizes (e.g. Cohen's *d*, Pearson's *r*), indicating how they were calculated

Our web collection on statistics for biologists contains articles on many of the points above.

Software and code

Policy information about availability of computer code

Data collection	Personal identifiers were encrypted by a third-party system overseen by the Icelandic Data Protection Authority<br>Attune Nxt version 3.2.1 was used to collect flow cytometry data
Data analysis	HLA alleles were genotyped using GraphTyper<br>NetMHCpan v4.0 server https://services.healthtech.dtu.dk/service.php?NetMHCpan-4.0 was used to predict binding of SARS-CoV-2 derived peptides to any of the significantly associating MHC alleles<br>ConSurf server https://consurf.tau.ac.il/ was used to determine conservation patterns and scores of the S and N proteins of SARS-CoV-2<br>FlowJo software version 10.7.1 was used for analyses of flow cytometry data and as a comparison to the automatic gating.<br>R script was designed for automatic gating of flow cytometry data. This script will be made publicly available upon publication.<br>R was used to analyze data and create plots

For manuscripts utilizing custom algorithms or software that are central to the research but not yet described in published literature, software must be made available to editors and reviewers. We strongly encourage code deposition in a community repository (e.g. GitHub). See the Nature Portfolio guidelines for submitting code & software for further information.

Data

Policy information about availability of data

All manuscripts must include a data availability statement. This statement should provide the following information, where applicable:

- Accession codes, unique identifiers, or web links for publicly available datasets
- A description of any restrictions on data availability
- For clinical datasets or third party data, please ensure that the statement adheres to our policy

The datasets generated during and/or analysed during the current study are available from the corresponding author on reasonable request

Field-specific reporting

Please select the one below that is the best fit for your research. If you are not sure, read the appropriate sections before making your selection.

☒ Life sciences ☐ Behavioural & social sciences ☐ Ecological, evolutionary & environmental sciences

For a reference copy of the document with all sections, see nature.com/documents/nr-reporting-summary-flat.pdf

Life sciences study design

All studies must disclose on these points even when the disclosure is negative.

Sample size	We recruited 759 convalescent SARS-CoV-2 cases and 491 uninfected controls which represents the largest study on SARS-CoV-2 reactive T-cell responses in convalescent SARS-CoV-2 cases and uninfected individuals.
Data exclusions	Based on the mean CD4+ and CD8+ T-cell counts of all participants with the 6 stimulation types tested, we defined a QC threshold of 20,000 cells and 10,000 cells as cut-off for CD4+ T and CD8+ T cell counts, respectively. Based on this threshold, 4 and 12 samples were excluded from CD4+ and CD8+ T cell analysis, respectively.
Replication	A literature search was performed to validate our findings in other smaller and previously reported research. In line with previous reports we observed robust CD4+ T-cell responses following SARS-CoV-2 infections to the whole S protein, S1 subunit , M and N proteins as well as the CD8+ T cell responses were more modest. Further, we observed stable SARS-CoV-2 reactive CD4+ and CD8+ T cell responses for up to eight months from infection in agreement with previous reports. The strength of our study lies in the large number of SARS-CoV-2 cases and uninfected controls that allowed us to observe novel associations between T cell responses and class I alleles, disease severity, age and humoral responses that smaller studies have not been powered to address. These novel associations remain to be replicated in other cohorts.
Randomization	Randomization was not used. We collected as many samples as we could from cases of the first SARS-CoV-2 wave in Iceland and invited uninfected controls of similar age as the cases. We measured antibody levels using three pan-Ig antibody tests and required at least two out of three to be positive for an individual to be considered a SARS-CoV-2 case. Control samples collected in 2020 were required to be negative for all three antibody assays.
Blinding	Investigators were blinded during data collection and initial analyzes

Reporting for specific materials, systems and methods

We require information from authors about some types of materials, experimental systems and methods used in many studies. Here, indicate whether each material, system or method listed is relevant to your study. If you are not sure if a list item applies to your research, read the appropriate section before selecting a response.

Materials & experimental systems

n/a	Involved in the study	
☐	☒	Antibodies
☒	☐	Eukaryotic cell lines
☒	☐	Palaeontology and archaeology
☒	☐	Animals and other organisms
☐	☒	Human research participants
☒	☐	Clinical data
☒	☐	Dual use research of concern

Methods

n/a	Involved in the study	
☒	☐	ChIP-seq
☐	☒	Flow cytometry
☒	☐	MRI-based neuroimaging

Antibodies

Antibodies used	Ultra-LEAF™ Purified anti-human CD28 Antibody Biolegend #302934<br>Ultra-LEAF™ Purified anti-human CD49d Antibody Biolegend #304340

Human TruStain FcX™ Biolegend #422302
APC/Cyanine7 anti-human CD3 Antibody Biolegend #300318
Brilliant Violet 605™ anti-human CD4 Antibody Biolegend #300556
PE/Cyanine7 anti-human CD8a Antibody Biolegend #301012
FITC anti-human IFN-γ Antibody Biolegend #502506
APC anti-human TNF-α Antibody Biolegend #502912
PE anti-human IL-2 Antibody Biolegend #500307

Validation

Ultra-LEAF™ Purified anti-human CD28 Antibody Biolegend #302934 (lot B313286). Monclonal antibody against human CD28.Reactivity: Human, Baboon, Capuchin Monkey, Chimpanzee, Cynomolgus, Pigtailed Macaque, Rhesus, Sooty Mangabey, Squirrel Monkey. This lot of antibody was quality control tested by immunofluorescent staining with flow cytometric analysis by the manufacturer.
Ultra-LEAF™ Purified anti-human CD49d Antibody Biolegend #304340 (lot B301643). Monclonal antibody against human CD28. Reactivity:Human, African Green, Baboon, Cat (Feline), Cattle (Bovine, Cow), Chimpanzee, Common Marmoset, Cynomolgus, Dog (Canine), Horse (Equine), Rhesus, Sheep (Ovine), Squirrel Monkey. Purified by affinity chromatography and each lot of this antibody was quality control tested by immunofluorescent staining with flow cytometric analysis by manufacturer.
Human TruStain FcX™ Biolegend #422302 (lot B327747). This product lot has passed BioLegend's Flow cytometry QC testing.
APC/Cyanine7 anti-human CD3 Antibody Biolegend #300318 (lot B319959). Monoclonal antibody against human CD3. Reactivity, human. Each lot of this antibody was quality control tested by immunofluorescent staining with flow cytometric analysis, by the manufacturer.
Brilliant Violet 605™ anti-human CD4 Antibody Biolegend #300556 (lot B316677). Monoclonal antibody against human CD4. Reactivity, Human, Chimpanzee. Each lot of this antibody was quality control tested by immunofluorescent staining with flow cytometric analysis by manufacturer.
PE/Cyanine7 anti-human CD8a Antibody Biolegend #301012 (lotB308784). Monoclonal antibody against human Cd8a. Reactivity, Human, Cross-Reactivity: Chimpanzee, Baboon, Cynomolgus, Rhesus, Pigtailed Macaque, Sooty Mangabey. each lot of this antibody is quality control tested by immunofluorescent staining with flow cytometric analysis by manufacturer.
FITC anti-human IFN-γ Antibody Biolegend #502506 (lot B317380). Monoclonal antibody against human IFN-γ. ReactivityHuman, Cross-Reactivity: Chimpanzee, Baboon, Cynomolgus, Rhesus. Each lot of this antibody is quality control tested by intracellular immunofluorescent staining with flow cytometric analysis by manufacturer.
APC anti-human TNF-α Antibody Biolegend #502912 (lot B311747). Monoclonal antibody against human TNF-α. ReactivityHuman, Cat (Feline) Cross-Reactivity: Chimpanzee, Baboon, Cynomolgus, Rhesus, Pigtailed Macaque, Sooty Mangabey, Swine (Pig, Porcine). Each lot of this antibody is quality control tested by intracellular immunofluorescent staining with flow cytometric analysis by manufacturer.
PE anti-human IL-2 Antibody Biolegend #500307 (lot B314271). Monoclonal antibody against human IL-2. ReactivityHuman, Cat (Feline) Cross-Reactivity: Chimpanzee, Baboon, Cynomolgus, Rhesus, Sooty Mangabey. Each lot of this antibody is quality control tested by intracellular immunofluorescent staining with flow cytometric analysis by manufacturer.

Human research participants

Policy information about studies involving human research participants

Population characteristics

The mean age of SARS-CoV-2 cases was 43.9 years (15.3 s.d.), pre-pandemic controls 55.5 years (11.2 s.d.) and controls collected during the pandemic 55 years (14.9 s.d.). 53% of the SARS-CoV-2 cases were females, 59% were females in the pre-pandemic and in controls collected during the pandemic

Recruitment

We recruited 759 convalescent SARS-CoV-2 cases and 491 uninfected controls. For 148 we had collected samples before the pandemic (October, 2001-February, 2020). Eighty of the pre-pandemic samples were from SARS-CoV-2 cases from whom we also had samples following the infection (May-December, 2020) and 44 were from controls from whom we also had samples collected during the pandemic (June-December, 2020). For 90 of the cases we collected paired samples during the pandemic.

Ethics oversight

The study was approved by the National Bioethics Committee (VSN_20-076) after review by the Icelandic Data Protection Authority (DPA). Personal identifiers were encrypted by a third-party system overseen by the DPA and all processing of personal data was in agreement with conditions set by the DPA (PV_2017060950ÞS).

Note that full information on the approval of the study protocol must also be provided in the manuscript.

Flow Cytometry

Plots

Confirm that:

☒ The axis labels state the marker and fluorochrome used (e.g. CD4-FITC).

☒ The axis scales are clearly visible. Include numbers along axes only for bottom left plot of group (a 'group' is an analysis of identical markers).

☒ All plots are contour plots with outliers or pseudocolor plots.

☒ A numerical value for number of cells or percentage (with statistics) is provided.

Methodology

Sample preparation

Peripheral blood mononuclear cells (PBMCs) were isolated from venous blood samples via standard Ficoll-Paque density gradient centrifugation at 800G for 15 min in 50ml Blood-Sep spin tubes and cryopreserved in liquid nitrogen. Prior to use

cells where thawed incubated over night at 37°C and 5% CO_2 at 1.5E7 cells/mL in RPMI 1640 supplemented with 10% fetal bovine serum (FBS) and 1x Penicillin-Streptomycin. After resting over night cells were filtered, counted and resuspended at 1E7 cells/mL. Cells were stimulated 0.5μg/mL SARS-CoV-2 Prot_M, _N, _S, _S1 or CMV peptides pools along with 0.3μg/mL CD28 and CD49d antibodies co-stimulation for 8h total, 5μg/mL Brefeldin A and Monensin were added at 5 μg/mL after 30 min.
Cells were washed in phosphate-buffered saline (PBS), Fc receptors blocked with TruStain FcX and viable cells identified by exclusion using LIVE/DEAD Fixable Aqua Dead Cell Stain Kit. Cells were washed again with PBS supplemented with 2% FBS (FACS buffer) and surface markers were detected via addition of directly conjugated CD3-APC-Cy7, CD4-BV605 and CD8-PE-Cy7 antibodies at pre-titrated concentrations for 20 min at room temperature. Cells were then washed again in FACS buffer and fixed/permeabilized according to manufacturer's instructions using a FoxP3/Transcription Factor Staining Buffer Set.
Intracellular markers were detected via the addition of directly conjugated IFNγ-FITC, TNFα-APC and IL-2-PE antibodies at pre-titrated concentrations in FACS buffer for 20 min at room temperature.

Instrument

Attune NxT, Acoustic Focusing Cytometer. Model AFC2

Software

Attune Nxt version 3.2.1 was used to collect flow cytometry data
FlowJo software version 10.7.1 was used for analyses of flow cytometry data and as a comparison to the automatic gating.
R script was designed for automatic gating of flow cytometry data. This script will be made publicly available upon publication.

Cell population abundance

Based on the mean CD4+ and CD8+ T-cell counts of all participants with the 6 stimulation types tested, we definedd a QC threshold of 20,000 cells and 10,000 cells as cut-off for CD4+ T and CD8+ T cell counts, respectively. The mean CD4+ T cell count passing the CD4+ T cell criteria was 69,367 cells in cases, 64,903 in pre-pandemic controls and 69, 098 in controls collected during the pandemic. The mean CD8+ T-cell count passing the CD8+ T-cell criteria was 33.330 in cases, 30,148 in pre-pandemci controls and 32,084 in controls collected during the pandemic. After accounting for age and sex, the pre-pandemic samples had on average 9.0% (95% CI: 4.5%, 13.2%) fewer CD4+ T-cells than the samples collected during the pandemic, likely reflecting the negative effect of sample storage on the CD4+ T-cell count . We saw no significant difference in CD8+ T-cell counts between samples collected before and during the pandemic. There was no difference in total CD4+ or CD8+ T-cell counts between cases or controls after accounting for age and sex

Gating strategy

Core gates included singlet isolation (FSC-H versus FSC-A), live CD3 selection (CD3 versus Aqua), lymphocyte enrichment (SSC-A versus FSC-A), CD4 or CD8 selection (CD4 versus CD8). Further gating focused on single, double and triple cytokine producing CD4+ and CD8+ T-cells. Further description of gating strategy is found in methods and Supplementary Information

☒ Tick this box to confirm that a figure exemplifying the gating strategy is provided in the Supplementary Information.
